# Energy metabolism and glutamate-glutamine cycle in the brain: a stoichiometric modeling perspective

**DOI:** 10.1186/1752-0509-7-103

**Published:** 2013-10-10

**Authors:** Francesco A Massucci, Mauro DiNuzzo, Federico Giove, Bruno Maraviglia, Isaac Perez Castillo, Enzo Marinari, Andrea De Martino

**Affiliations:** 1 Departament d’Enginyeria Quimica, Universitat Rovira i Virgili, 43007 Tarragona, Spain; 2 Magnetic Resonance for Brain Investigation Lab, Enrico Fermi Center, Roma, Italy; 3 Dipartimento di Fisica, Sapienza Università di Roma, P.le Aldo Moro 2, 00185 Roma, Italy; 4 Fondazione Santa Lucia, Roma, Italy; 5 Department of Mathematics, King’s College London, Strand, London, WC2R 2LS, UK; 6 Center for Life Nano Science@Sapienza, Istituto Italiano di Tecnologia, Viale Regina Elena 291, 00161 Roma, Italy; 7 CNR–IPCF, Unità di Roma Sapienza, Roma, Italy

**Keywords:** Brain energetics, Lactate shuttle, Metabolic modeling, Glutamate-glutamine cycle, Glucose partitioning, OGI

## Abstract

**Background:**

The energetics of cerebral activity critically relies on the functional and metabolic interactions between neurons and astrocytes. Important open questions include the relation between neuronal versus astrocytic energy demand, glucose uptake and intercellular lactate transfer, as well as their dependence on the level of activity.

**Results:**

We have developed a large-scale, constraint-based network model of the metabolic partnership between astrocytes and glutamatergic neurons that allows for a quantitative appraisal of the extent to which stoichiometry alone drives the energetics of the system. We find that the velocity of the glutamate-glutamine cycle (*V*_cyc_) explains part of the uncoupling between glucose and oxygen utilization at increasing *V*_cyc_ levels. Thus, we are able to characterize different activation states in terms of the tissue oxygen-glucose index (OGI). Calculations show that glucose is taken up and metabolized according to cellular energy requirements, and that partitioning of the sugar between different cell types is not significantly affected by *V*_cyc_. Furthermore, both the direction and magnitude of the lactate shuttle between neurons and astrocytes turn out to depend on the relative cell glucose uptake while being roughly independent of *V*_cyc_.

**Conclusions:**

These findings suggest that, in absence of *ad hoc* activity-related constraints on neuronal and astrocytic metabolism, the glutamate-glutamine cycle does not control the relative energy demand of neurons and astrocytes, and hence their glucose uptake and lactate exchange.

## Background

Sustained cerebral activity is crucially dependent on the functional and metabolic interplay of neurons and glial cells. Spectroscopic and imaging methods have indeed shown that the brain accommodates a wealth of cell-to-cell interactions, which ultimately have contributed to displace the decades-old notion that merely coupled whole brain activity to neuronal glucose oxidation (for a comprehensive review, see [1]). In particular, carbohydrate metabolism is compartmentalized among neurons and astrocytes, which, together with the interstitial space, represent nearly 90% of the tissue. Although there is evidence for the trafficking of metabolic intermediates between the two cell types, its significance and dependence on the activation state are not fully elucidated. More than 15 years ago it was hypothesized that astrocytes may support the energetics of brain function by the provision of glucose-derived lactate to neurons, in an activity-dependent manner [2]. However, the idea of a metabolically significant astrocyte-to-neuron lactate shuttle (ANLS), as well as the activity-dependent increase in astrocytic glucose uptake, has proven to be rather difficult to confirm *in vivo*, while indirect and not always reproducible experimental proof was mainly obtained from experiments on cell cultures (see [3,4] and the excellent reviews [5,6]).

The difficult interpretation and integration of the experimental findings produced a substantial theoretical effort aimed at characterizing intra- and inter-cellular metabolic fluxes [7-12]. So far, mathematical models of transport and metabolism of glucose in neurons and astrocytes using either kinetic [7,9-11] or stoichiometry-based [13-15] approaches have provided conflicting results about the relevance of the cell-to-cell lactate shuttle (CCLS) (see [16] for a recent review). This, in turn, raised some debate, especially concerning the partitioning of glucose between neurons and astrocytes and the potentially resulting intercellular lactate flow [17]. A recent flux-balance-analysis (FBA) study indicates that the direction and magnitude of the CCLS between neurons and astrocytes depends critically on the relative uptake of blood-borne glucose [18]. The sharing of glucose between the two cell types is itself governed by the internal energetic demand of cells, implying that glucose partitioning alone cannot be used to draw any conclusion on the functional variations of the CCLS [11]. A critical reassessment of previous modeling results suggests that the CCLS might remain of minor significance in terms of transferred carbon equivalents [19].

On the other hand, the known regulations of enzyme-catalyzed reactions implemented in dynamical models have so far proved insufficient to justify a fundamental energetic role for the CCLS [11]. In particular, the differences between metabolic pathways of neuronal and astrocytic networks do not imply the occurrence of lactate exchange between cells, most likely because neurons and astrocytes do possess a relatively high self-sufficiency for both glycolytic and oxidative glucose metabolism (see [20] and references therein). This means that lactate is oxidatively metabolized in the same compartment where it is produced by glycolytic processing of glucose.

The aim of this study is to examine the activity-dependent metabolic cooperation of glutamatergic neurons and astrocytes from a network-based perspective. Specifically, two issues lie at the core of our work: (i) the correlation between partitioning of glucose and lactate shuttling; and (ii) their functional modulation across varying levels of glutamate-glutamine cycle. We have employed a constraint-based setting where an extensive and controlled sampling of the solution space is possible [21,22] on a large-scale model of compartmentalized brain energy metabolism. At odds with previous studies employing constraint-based schemes to analyze the neuron-glia system in specific conditions (different from those considered here, see [23]), our approach does not rely on an objective function (which in our case would be hard to design) to define the relevant states. In addition, it allows to analyze in detail the feasible metabolic states for networks whose sizes are beyond those covered by other approaches like Bayesian Flux Balance Analysis (BFBA) [15] or Montecarlo sampling of mass-balance equations [24]. Finally, we have not made any special assumption on the regulation of biochemical pathways with respect to the activation level, nor have we imposed specific constraints on transport fluxes (except for the uptake of glucose that we use to fine-tune the oxygen-to-glucose index (OGI), see below). In short, we show that, within our stoichiometric approach: 

(a) the OGI is able to distinguish states characterized by different levels of neurotransmission, as flux configurations with larger OGIs typically carry smaller values for the velocity of the glutamate-glutamine cycle;

(b) the partitioning of glucose between neurons and astrocytes is roughly independent of the level of activity;

(c) the magnitude and direction of the CCLS depend strongly on glucose partitioning while being roughly independent of the level of neurotransmission.

In other terms, within a purely stoichiometric model, the system’s energetics is determined to a significant extent by the sharing of glucose. These results support the idea that neurotransmission does not impose significant constraints on glucose partitioning or CCLS. In addition, we show that (d) the overall degree of correlation among metabolic reaction fluxes between and within cells changes drastically in the presence of neurotransmission, pointing to an extended metabolic and possibly functional partnership between neurons and astrocytes.

## Methods

### Network reconstruction

**Figure 1:**
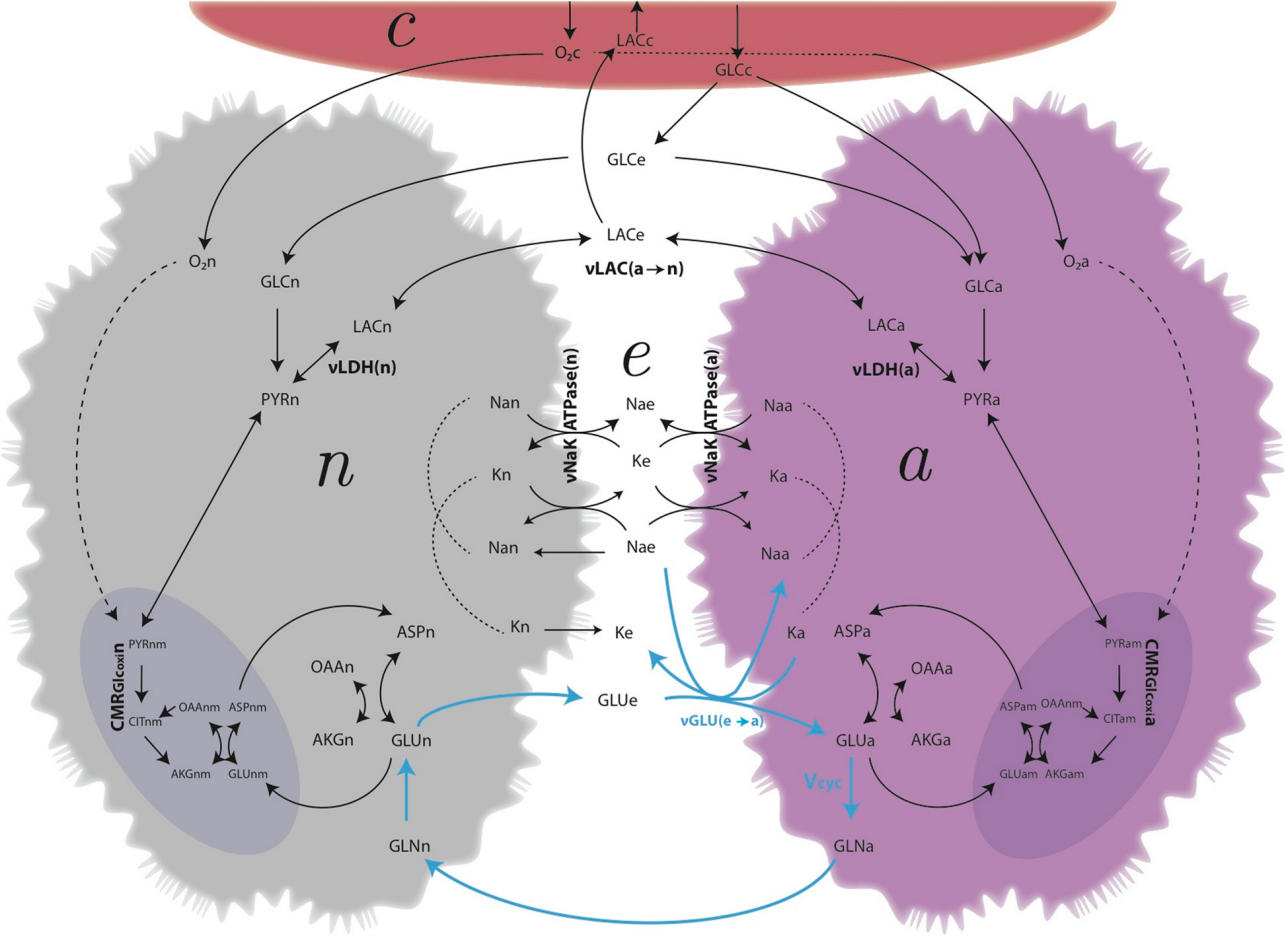
**Schematics of the model.** The figure shows selected pathways linking the four compartments of the model (capillary, interstitium or extracellular space, neuron, astrocyte). Nutrients from the blood capillary have to traverse endothelium and basal lamina (these elements have been lumped together with the capillary) to enter the brain parenchyma. Thus, arrows connecting directly capillary and cell interior represent flows across basal lamina after endothelium. Note that this shortcut makes sense for the diffusion of oxygen to neurons and astrocytes, as well as for the transport of glucose to the astrocytic compartment only. Indeed, astrocytes but not neurons are in close apposition to cerebral blood vessels. Most of the nutrients delivery to the brain occurs through interstitial space, which is therefore the primary common element for intercellular metabolite trafficking. Once into the cells, glucose (GLC) is metabolized via glycolysis to pyruvate (PYR), which can be either reduced to lactate (LAC) or further oxidized in the cell TCA cycle requiring oxygen (O_2_). Neuronal glutamate (GLU) is sequestered by the TCA cycle at the level of alpha-ketoglutarate (AKG) and loaded into synaptic vesicles (not shown). Neurotranmission evokes the release of vesicular glutamate into the extracellular space, from where it is taken up by astrocytes and mixed with their glutamate pool. Astrocytic glutamate can either be converted to glutamine (GLN) for export to neurons or enter the TCA cycle. The entire process consumes energy due to up-regulation of astrocytic Na^+^/K^+^-ATPase and glutamine synthetase (*V*_cyc_), as well as neuronal vesicle (re)filling. According to the minimal-constraints strategy employed in the present model, ionic fluxes in neurons via ligand- and voltage-gated ion channels and in astrocytes via Na ^+^/K ^+^ cotransporter follows neurotransmission passively (see text). See the Supporting Information for the full details of the network structure (139 reactions among 108 different chemical species).

The reaction network we considered (a reduced sketch of which is given in Figure 1) is composed of four main compartments: neuron (n), astrocyte (a), extracellular space (e) and blood capillary (c). Within the neuronal and astrocytic elements we also distinguished the cytosol (nc and ac, respectively), mitochondria (nm and am) and synaptic vesicles (nv, only in neurons). Transport of nutrients from the blood to the brain parenchyma is provided by the capillary. We assumed that under resting conditions glucose and oxygen irreversibly enter the brain, while lactate is not significantly exchanged [25,26]. Specifically, glucose can be taken up directly by astrocytes via the basal lamina or can diffuse into the extracellular space [9]. The latter, in turn, is a common compartment for glucose uptake by neurons and astrocytes, as well as for lactate shuttling between the two cell types. Oxygen can freely diffuse from the capillary to cells. We lumped together the endothelium and basal lamina with the capillary compartment, which also means that we assume a negligible metabolism for endothelial cells.

The neuronal and astrocytic compartments are equipped with the enzymatic machinery to carry out the main pathways of carbohydrate metabolism (glycolysis, pentose phosphate shunt, TCA cycle, oxidative phosphorylation) [27]. Both cell types indirectly transport reducing equivalents (i.e. NADH) from cytosol to mitochondria via malate-aspartate shuttle (MAS) (see [28]). We made the simplifying assumption that only astrocytes are capable of glutathione synthesis because neurons, unlike astrocytes, are unable to efficiently transport cystine [29] and, importantly, they cannot increase the substrate flow through glutamate-cystine ligase [30], the rate limiting step in glutathione synthesis. Yet, the antioxidant system is equally present in neurons and astrocytes to detoxify the reactive oxygen species (ROS) produced by oxidative phosphorylation. The stoichiometry of ROS production by oxidative phosphorylation was chosen assuming that 5% to 15% of glucose is processed through the pentose phosphate pathway to regenerate the NADPH required for reducing oxidized glutathione [29,30]. Anaplerosis of TCA cycle intermediates is performed by pyruvate carboxylation, which is confined to astrocytes [31], as well as by the activity of the neuronal and astrocytic malic enzyme [32].

The functional portion of the metabolic network includes glutamatergic neurotransmission, transmitter recycling and ionic movements, that together establish the coupling between activity and metabolism through the action of the Na/K-ATPase [33]. Specifically, the glutamate stored in neuronal synaptic vesicles can be released in the extracellular space, from where it is taken up by astrocytes in co-transport with three Na^+^ ions and counter-transport of one K^+^ ion. Glutamate is amidated to glutamine by astrocytic glutamine synthetase (GS) with the concurrent hydrolysis of one molecule of ATP. Glutamine is then exported to neurons where it is eventually converted back to glutamate and loaded into synaptic vesicles again, which costs another ATP. Astrocytic uptake of glutamate and release of glutamine, together with neuronal uptake of glutamine and release of glutamate configure the so-called glutamate-glutamine cycle. In this way, the clearance of neuronally released glutamate from the extracellular space is mostly accomplished by astrocytes [34], although a fraction of the neurotransmitter can be taken up by neurons, especially in synapses not associated with astrocytic processes [35]. At odds with previous mass-balance modeling works [15,17,18], we included the ionic currents related to membrane depolarization, albeit these were not explicitly linked to glutamate release. In particular, neurons possess Na^+^ and K^+^ channels that mimic voltage-gated ion channels and astrocytes can also take up potassium from the extracellular space with the Na-K cotransporter. Overall, the fluxes of Na^+^ and K^+^ activate Na/K-ATPase, which consumes one ATP to transport three Na^+^ out of the cell and two K^+^ inside the cell. Importantly, not all the glutamate which is taken up by astrocytes is channeled via the glutamate-glutamine cycle. Glutamate in astrocytes can be used for energy production by entering the TCA cycle after conversion to alpha-ketoglutarate through transamination by aspartate aminotransferase (AAT) or dehydrogenation by glutamate dehydrogenase (GDH) [36]. We did not include the action of other transaminase, e.g. alanine aminotransferase. This choice precludes testing the exchange of lactate and of alanine between neurons and astrocytes for maintaining ammonia homeostasis during glutamate-glutamine cycle [37]. However, the role of this shuttle was experimentally found to be activity-independent in neuronal-astrocytic cultures [38]. Finally, we conformed to other mass-balance modeling works [15,17] in excluding the pathways involved in the synthesis and degradation of nucleic and amino acids. This is justified by the different characteristic time-scales of processes underlying energy metabolism and gene expression, and does not rule out the possibility of any change in flux velocity brought about by e.g. protein translocation.

The network altogether consists of 139 reactions processing 108 different chemical species. The full lists of reactions and chemical species is reported in the Additional file 1: Supporting Text.

### Flux model

We assume that the reaction network described above operates at stationarity, i.e. that reaction fluxes in feasible configurations are constant. More precisely, we postulate that the system is kept in a non-equilibrium steady-state (NESS) by the boundary conditions (in our case, by the fluxes of glucose and oxygen into the capillary). Although a steady-state approach for cerebral metabolism will clearly be unable to capture transient or kinetic effects, it can be justified by several considerations. In first place, a typical experiment is performed on tissue volumes containing a large number of cells, and a standard outcome will roughly represent an average over cells in the entire sample. Such averaging can be reasonably approximated with a steady–state assumption, provided the environmental conditions, including stimulation and activation, are stationary. This excludes from the analysis the time intervals associated to the transitions from one state to another (e.g. stimulation onset), which commonly last for a few tenths of a second before a steady–state is attained [39]. Related to this is the fact that in many cases the duration of a stimulus largely exceeds the equilibration time of metabolite concentrations. It has indeed been shown that sustained stimulation induces, after a short transient, a switch to different stationary states for metabolism, neuronal activity, and hemodynamic responses [39-41]. Essentially, the steady-state approach allows for the study of brain metabolism on a time scale lying between the fast adaptation to the change in the activation condition and the slow adjustment of regulatory mechanisms. Finally, within this approach it is possible to treat systems much larger than those accessible to kinetic modeling (see for example [23]), where only a few nodes of the metabolic networks are usually included.

Constraint-based models provide a standard framework for the analysis of biochemical networks in NESS. In Flux-Balance Analysis (FBA), for instance, one imposes that the vector of concentrations of intracellular metabolites **c**, which in general would vary in time according to

(1) $$\dot{c}=S\nu -b,$$

(where **S** denotes the *M* × *N* stoichiometric matrix, *N* is the number of reactions, *M* that of metabolites, ***ν*** the vector of reaction fluxes, and **b** the vector of in- and out-takes that govern the transport of chemical species to and from the system) is constant. In turn, fluxes need to adjust to satisfy simple mass-balance conditions for the individual chemical species, amounting to the set of *M* equations

(2) Sν=b.

Note that the elements of **b** are non-zero only for metabolites that are exchanged with the environment. For sakes of definiteness, bounds of variability for each flux *ν*_*i*_ (*i* = 1,…,*N*) need to be specified. Usually, such bounds account for reversibility assignment, i.e. they are either of the form -*∞* < *ν*_*i*_ < *∞* (for reversible reactions) or of the form 0 ≤ *ν*_*i*_ < *∞* (for irreversible reactions), although in some cases physiological considerations may lead to consider more complicated cases, e.g. *ν*_0_ ≤ *ν*_*i*_ < *∞* with *ν*_0_ > 0. (In the present study, we shall only consider bounds for the putative reversibility of reactions, as detailed in the network reconstruction reported in the Additional file 1: Supporting text, except for the glucose uptake flux to the capillary which is taken to be fixed. See below for details.)

The system (2) now defines a solution space as a polytope of dimension *N* - *M* (or more precisely, *N* - rank(**S**); note that, typically, *N* > *M*). In the absence of a refinement criterion, like an *ad hoc* optimization prescription (see e.g. [42] for an excellent introduction to this modeling perspective), the set of solutions should ideally be sampled uniformly to extract both the individual solutions as well as the statistics of fluxes (averages, correlations, etc.). This is indeed the type of information we are interested in retrieving in the present case. Unluckily, exact sampling algorithms (e.g. Monte Carlo) are still inapplicable to genome-scale flux models when the dimension of the solution space exceeds a few tens because of their high computational costs [43]. (See however [44] for a promising set of alternative techniques.) In addition, the straightforward application of FBA-type of constraints in the our case is also made difficult by the fact that our reconstruction is largely incomplete. This means that the pathways we do not include may have a considerable cross-talk with the core carbon pathways on which we focus, so that the solutions of (2) might depend strongly on the choice of the boundary fluxes that represent the interaction between pathways included in the reconstruction and the rest of the metabolic network.

We therefore took a step back with respect to FBA and considered a broader type of conditions, inspired by Von Neumann’s model of production networks [45]. In essence we simply replace (2) with

(3) Sν≥0

for all intracellular metabolites, while keeping (2) for in-takes (nutrients). Clearly, the main difference with (2) is that (3) allows for flux vectors generating a net production of chemical species (corresponding to the metabolites for which the strict inequality holds in (3)). In a nutshell, the rationale behind this is that a net production of certain compounds might be expected in cells if they need to be employed in macromolecular processes (e.g. proteinogenesis) lying out of the domain of metabolism or, more relevantly to our case, in portions of the network that are not included in the reconstruction. (In other terms, the presence of gaps and their impact on the flux organization of the core network may be smoothened out by softening the constraints.) Therefore, strictly speaking, a NESS where the concentration of certain metabolites is formally increasing in time (corresponding to the positive components of the vector **S*ν***) can be physiologically viable. Once the nutrient availability is fixed through the boundary fluxes, the cell’s metabolic production and nutrient usage profiles can be determined self-consistently from the solutions of (3).

The solutions of (2) and (3) will obviously coincide if all inequalities in (3) become equalities, though in general this does not need to be the case. The main technical advantage of using (3) lies in the existence of an effective and scalable relaxation algorithm that allows to obtain a statistically controlled sampling of its solution space in very modest CPU times. Such a method has been defined in [46] and employed in e.g. [21] and [22] to analyze the metabolic capabilities and the energy balance of the bacterium *E. coli*. The statistical properties of the solution space sampling thus obtained are discussed in [22] and further explained in the Additional file 1: Supporting text. In brief, the algorithm makes use of a prior probability distribution of fluxes to initialize the flux variables and generates solutions such that the *average* Euclidean distance between the solutions and the prior is minimized, the average being carried out over initial flux states sampled from the prior. A sufficiently unbiased prior (e.g. a set of uncorrelated uniform distributions, as we have employed here) then injects minimal *a priori* information in the solution space and therefore provides a reasonably unbiased, statistically controlled sampling of the feasible flux states of the network. In turn, such an information allows to extract physiological details of individual solutions, as well as statistical properties of the solution space (e.g. probability distributions of fluxes and correlations). This is the calculation scheme we have employed. Further details about the network (i.e. the matrix **S**), the flux model and the algorithm used in the present work are found in the Additional file 1: Supporting text.

In the following we shall denote the flux of an intracellular reaction or of a transport process respectively by the acronym of the corresponding enzyme or the name of the transported metabolite. We shall also highlight the compartment in which the reaction occurs (so that e.g. *ν*PDH(*n*) will stand for the pyruvate dehydrogenase–catalyzed reaction taking place in the neuron) or the source/destination compartments that are involved in a transport (for instance, *νO*_2_(*c* → *a*) will denote the transport of molecular oxygen from the capillary to the astrocyte). Shorthands like ν*O*_2_(→ *c*) will instead represent the supply of metabolites (oxygen in this case) to the capillary. Unless otherwise stated, fluxes are expressed in arbitrary units and error bars correspond to one standard error.

## Results

### Validation of the model: activation states

In the network model we consider, capillaries are supplied with two compounds, namely glucose and oxygen, which can then be transferred to the other compartments. We have characterized the metabolic activity of the brain by fixing only the uptake of glucose to the capillary, νGlc(→ *c*), while leaving the oxygen influx free. In these conditions, each fixed value of νGlc(→ *c*) generates a different solution space for (3), where ν*O*_2_(→ *c*) fluctuates across solutions (i.e., across feasible flux configurations). This in turn yields, for each selected value of νGlc(→ *c*), a distribution of values for the oxygen-glucose index (OGI), defined as the ratio between cerebral metabolic rates of oxygen (${\text{CMR}}_{O_{2}}$) and glucose (CMR_Glc_). As both nutrients do not accumulate in the tissue at steady-state, the OGI can be defined as

(4) $$\text{OGI}=\frac{{\text{CMR}}_{O_{2}}}{{\text{CMR}}_{\text{Glc}}}=\frac{\nu O_{2}(\to c)}{\nu \text{Glc}(\to c)}.$$

where nutrient influxes (both the numerator and the denominator) obey, as said above, mass-balance conditions:

(5) $$\nu O_{2}(\to c)=\nu O_{2}(c\to a)+\nu O_{2}(c\to n)\;$$

(6) νGlc(→c)=νGlc(c→a)+νGlc(c→e).

Note that the last equation does not involve the neuronal compartment because glucose enters neurons via the extracellular space only (as said before, the endothelium and basal lamina, which mediate the transport of glucose to astrocytes and extracellular space, are included in the capillary compartment). Note also that at steady-state one has

(7) νGlc(c→e)=νGlc(e→a)+νGlc(e→n).

Generically, larger OGIs imply larger fluxes through aerobic pathways, with OGI=6 as the physiologic maximum value for the steady state aerobic oxidation of glucose (corresponding to the fact that 6 oxygen molecules are required to metabolize glucose to water and carbon dioxide). Nevertheless, OGI values larger than 6 are possible in cells whenever the carbon supply for cellular respiration exceeds glucose processing through glycolysis (as happens, for instance, during lactate uptake from the bloodstream). In the flux model (3) it is possible to obtain OGI values slightly above 6, as a consequence of the fact that the oxygen intake to the capillary is a free variable, not bounded (within the model) by the condition OGI ≤ 6. From a modeling viewpoint, this may correspond to a small accumulation of intracellular oxygen due to e.g. the absence of some oxygen-consuming pathways in the network (this condition might not have a physiological counterpart). Experimental in vivo measurements show that the OGI decreases with increasing cerebral activation, from values around 5.5 (almost complete glucose oxidation) under awake resting conditions to values generally ranging from 4 to slightly above 5 during focal brain activity, depending for example on the stimulation paradigm, on the involved brain area, on the experimental procedure (reviewed in [47,48]). Since the contribution of individual physiologic processes under different conditions is not known, we sought to model the level of activation by using the OGI as a proxy. This assumption stems from the notion that different metabolic states can be characterized in term of their energy expenditure [49]. Furthermore, the transition to a more glycolytic than oxidative metabolism is thought to identify the transition from basal to activated conditions [50]. These arguments suggests that brain metabolism approaches full glucose oxidation as the overall signaling activity decreases.

**Figure 2:**
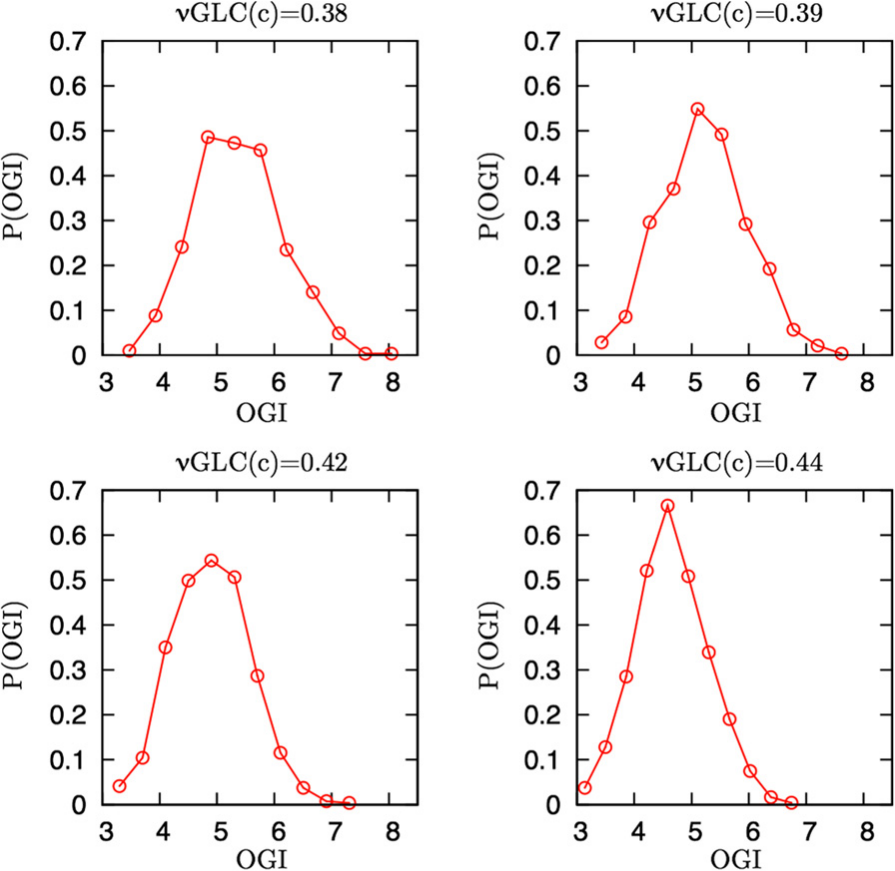
**Computed probability distributions of oxygen-to-glucose index (OGI) at different glucose uptakes.** Top left to bottom right: Uncoupling between glucose and oxygen utilization increases for increasing overall glucose uptake (i.e. enhanced glutamatergic activity). The mean OGI decreases from 5.5 to 4.5 in correspondence of an increased glucose consumption of about 15%. This behavior is in a qualitative agreement with experimental evidence, and allows for a definition of different activation states based on the uncoupling between glucose and oxygen consumption. Each OGI distribution thus identifies a subset of solutions for subsequent flux analysis.

We have hence solved (3) for different values of νGlc(→ *c*) recording the resulting OGI distributions. Figure 2 shows four distributions of OGI corresponding to different glucose consumption rates, starting from lower values of νGlc(→ *c*) corresponding to a larger average OGI. It should be noted that there is not a clear consensus about the quantitative degree of OGI decrease during activity. Indeed, while a value under activation around 5.1 is suggested by several works [47,48], many others point towards lower values (see e.g. [51,52] and the recent [53]). Because of this, and because the OGI distributions we found at a given νGlc(→ *c*) are rather broad, we preferred to explore a relatively broad range of OGI values.

We have further characterized the model by monitoring the degree of activation in terms of the velocity of the glutamine synthetase (GS) catalyzed reaction. In our simulated network, this univocally represents the rate of the so–called glutamate–glutamine cycle *V*_cyc_, or

(8) $$V_{\text{cyc}}=\nu \text{GS}(a).$$

It should be noted that, in vivo, there is a residual rate of Gln synthesis unrelated to neurotransmitters cycling [54], but under physiological conditions νGS(*a*)≃*V*_cyc_[55], and thus we conformed to our general choice of neglecting neurotransmission–unrelated amino acids synthesis also in this specific case. The assumption that the residual rate of Gln synthesis is independent of glutamatergic neurotransmission is made also in the original experimental work that reported *V*_cyc_[54], and the relevant implications are discussed therein.

**Figure 3:**
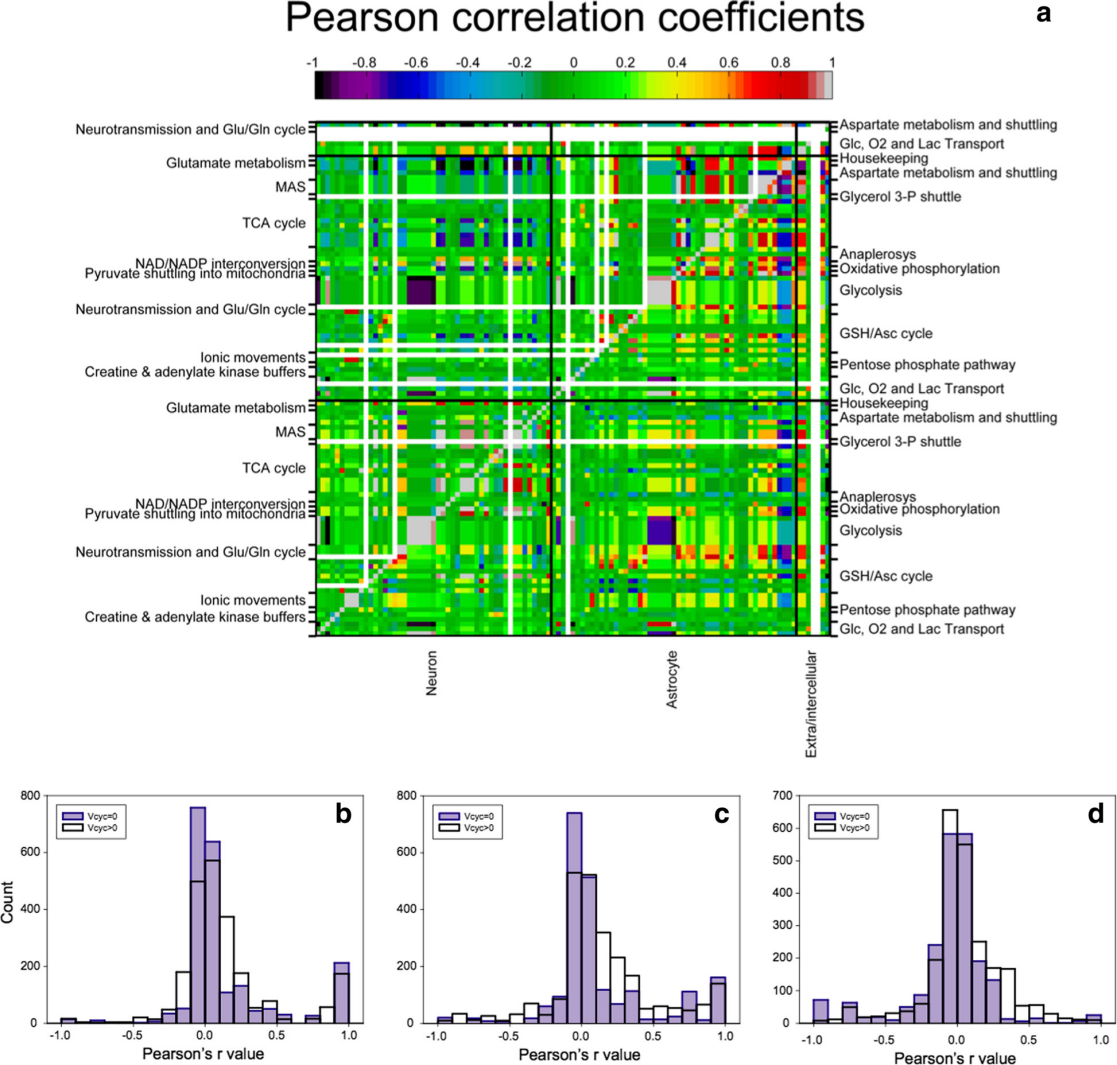
**Pearson correlation coefficients for each pair of reactions. (a)** Cycles, pathways and homogeneous reaction groups are reported sideways, the compartment is reported at the bottom. Neuron and astrocyte sector also include transports from/to the cell, while the extra/intercellular class groups reactions that either directly connect neuron to astrocyte, or involve only extracellular compartments. Null fluxes are represented in white. Above the diagonal we report the Pearson’s coefficients obtained by imposing *V*_cyc_ = 0, while below the diagonal *V*_cyc_ is allowed to assume positive values. **(b**,**c**,**d)** Histograms of the Pearson correlation coefficients for each pair of reactions within neurons **(b)**, astrocytes **(c)** or between cells **(d)**. For each plot, the distributions are reported for *V*_cyc_ = 0 (in pale blue) and *V*_cyc_ > 0 (transparent). Null fluxes (i.e. those fluxes that are plotted in white in panel **(a)**) are excluded from the histograms. Apart of the apparent larger number of null fluxes with *V*_cyc_ = 0, the histograms show that the bins at higher correlation tend to be more populated at *V*_cyc_ > 0 than at *V*_cyc_ = 0, while the opposite holds for central bins (i.e. those bins with very low direct or inverse correlation).

As happens for the OGI, each choice of νGlc(→ *c*) leads to a distribution of values of *V*_cyc_. Generically, solutions with *V*_cyc_ = 0 (i.e. no neurotransmission^a^) will coexist with solutions carrying a non-zero level of activation for any choice of νGlc(→ *c*). In order to highlight the quantitative changes induced by activation, we performed a correlation analysis between the fluctuations of flow rates of reaction or transport pathways, first in all the sampled solutions (i.e. with *V*_cyc_ ≥ 0) and then in solutions with *V*_cyc_ > 0. It turns out (see Figure 3) that the transition from uncostrained neurotransmission to presence of neurotransmission has profound consequences on the flux correlations within and between cells. The solutions obtained for *V*_cyc_ > 0 underlie a substantial increase of both the global and the regional correlation level of the network. This is especially true for the correlation between neuronal and astrocytic metabolism, confirming that the condition *V*_cyc_ > 0 identifies the functional and metabolic partnership between the two cell types. This finding shows that intercellular signaling has a major role for the overall metabolic regulation at tissue level, constraining the catabolism of each cell in a concerted range (see Discussion).

**Figure 4:**
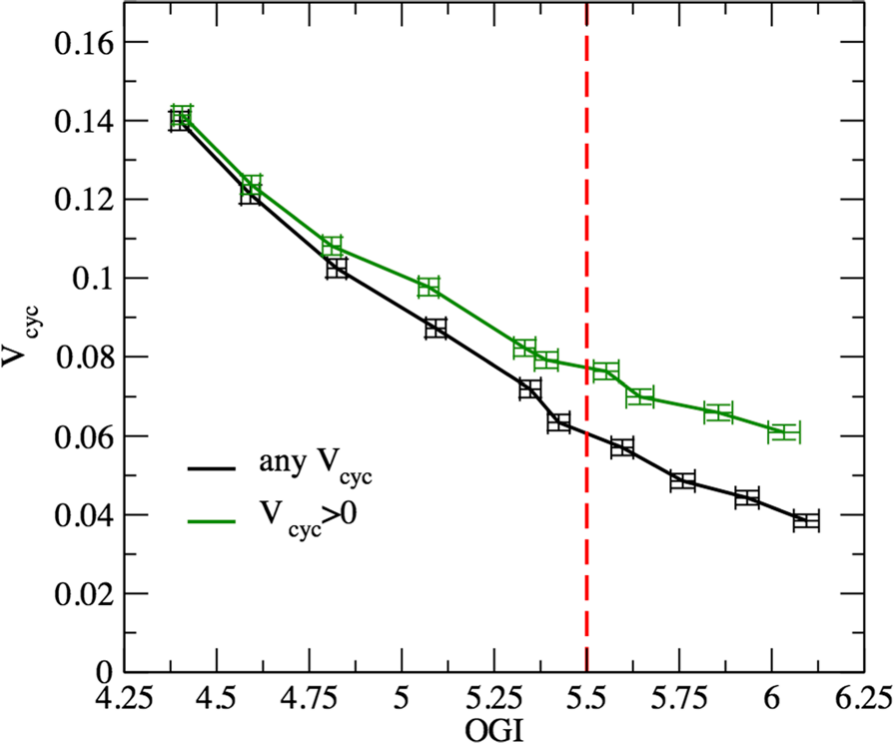
**Computed average rate of the glutamate-glutamine cycle versus average OGI.** Simulations show that the average *V*_cyc_ increases as the average OGI decreases, consistently with the underlying relationship between OGI and glucose uptake. The curves represent conditional averages, computed over solutions characterized respectively by *V*_cyc_ ≥ 0, and *V*_cyc_ > 0. The red dashed line identifies the awake resting state (OGI = 5.5). The crossing points between the line at OGI = 5.5 and each of the two curves identify the values of *V*_cyc_ relevant for the basal conditions. Specifically, *V*_cyc_ is roughly 0.06 or 0.08 for the groups *V*_cyc_ ≥ 0 and *V*_cyc_ > 0, respectively. The two curves are significantly different for OGI ≳4.75. This is consistent with the fact that the contribution of solutions with *V*_cyc_ = 0 becomes negligible at high activation levels.

The above results suggest that, in order to capture the quantitative changes that occur in solutions at higher levels of activation (recall that our model does not, per se, constrain the magnitude of neurotransmission), it is useful to analyze the behavior of the conditional average *V*_cyc_ versus the conditional average OGI. In Figure 4 we display the results obtained by retaining solutions with *V*_cyc_ ≥ 0 (i.e. all solutions, returning the standard average) and *V*_cyc_ > 0, respectively. One sees that as the average OGI decreases, the average *V*_cyc_ increases to an enhanced activation level. The slope of the curve is less negative for the condition *V*_cyc_ > 0, i.e. when the sample is restricted by filtering–out the states with suppressed neurotransmission. This changes the basal state at OGI = 5.5 to a rate of glutamate-glutamine cycle larger by about 30%, from 0.06 to 0.08 (roughly).

In summary, in agreement with the literature, we find that the rise in anaerobic glucose consumption during activation turns out to be out of proportion to oxygen utilization, as evidenced by the decrease in the (average) OGI for increasing values of *V*_cyc_ and CMR_Glc_. This indicates that the glutamate-glutamine cycle by itself suffices to explain part of the uncoupling between glucose and oxygen utilization. It should be emphasized that the reported OGI reduction, although potentially significant if caused by a specific subset of glycolytically-served energetic demands, does not change the overall strategy of brain energy metabolism, which remains largely aerobic because of the higher ATP yield of respiration [1].

### Neuronal oxidative glucose metabolism versus glutamatergic activity

**Figure 5:**
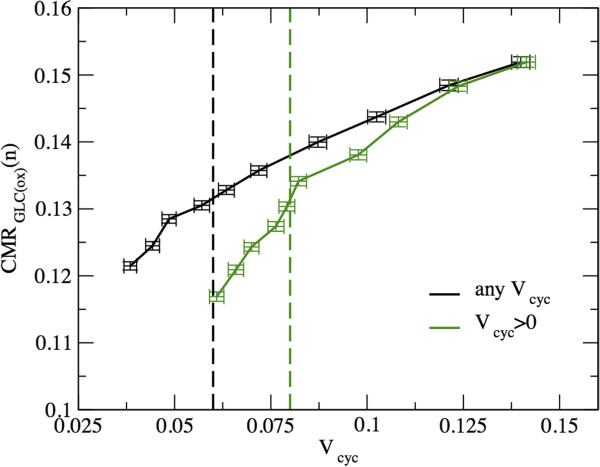
**Rate of glucose oxidation in neurons as a function of the Glu/Gln cycle. **The curves represent conditional averages, computed over solutions characterized respectively by *V*_cyc_ ≥ 0 and *V*_cyc_ > 0. The dashed lines identify the awake resting state (OGI = 5.5), corresponding to the group of solutions (*V*_cyc_ ≥ 0 and *V*_cyc_ > 0) plotted with the same color. For the condition *V*_cyc_ > 0, and possibly also for the condition *V*_cyc_ ≥ 0, the awake rest roughly corresponds to a change of the line slope. Thus, the transition from low to high activity is accompanied by a decreased energy consumption relative to what would be extrapolated at low activity levels. In particular, the slopes of the two curves at low *V*_cyc_ values are roughly 0.52 and 0.73 (for the groups *V*_cyc_ ≥ 0 and *V*_cyc_ > 0, respectively) and decrease to roughly 0.28 and 0.33 at high *V*_cyc_ values, suggesting a lack of energy demand at high activity (see text).

The relationship between glutamate-glutamine cycle and neuronal glucose oxidation was experimentally reported to be close to a 1:1 relation [56]. In particular, the rate of neuronal oxidative metabolism of glucose (i.e. the level of activity of pyruvate dehydrogenase, PDH) increases linearly with the velocity of transmitter cycling, with a slope close to one. Our framework allows to address the dependence of glucose oxidation in neurons on the rate of glutamate-glutamine cycle for various states of activation. Considering that PDH is the primary entry point of glucose-derived pyruvate into the TCA cycle, we define

(9) $${\text{CMR}}_{\text{Glc}(\mathrm{ox})}(n)=\frac{1}{2}\nu \text{PDH}(n),$$

where the factor 1/2 is required as glycolysis produces two molecules of pyruvate for each glucose molecule. Plotting the average CMR_Glc(ox)_(*n*) against the average *V*_cyc_ we find approximately two different regimes around the physiologic range corresponding to OGI ≃ 5.5 (see Figure 5). These regimes are characterized by almost linear relations, in agreement with the experimentally reported constant stoichiometry between aerobic Glc oxidation in neurons and glutamate cycling [54,56]. However, the coupling pattern changes as one explores states with values of *V*_cyc_ departing from the basal level to higher activity. The slope of the curve clearly increases if only strictly positive *V*_cyc_ are considered, although it stays below one. Interestingly, in the high *V*_cyc_ regime (upstream the awake value, for which no experimental data exist) the plot features a slight attenuation of the curve. The fact that the slope is lower than the linear extrapolation at high activity indicates that some source of energy consumption adds to the glutamate–glutamine cycle during the transition from normal to high neurotransmission levels. In this region, the discrepancy between simulated and experimental data is explained by the relatively low increase in ionic fluxes through neuronal voltage-gated Na^+^ and K^+^ channels obtained in our simulations (data not shown), suggesting that the glutamate–glutamine cycle alone is insufficient to account for the rise in brain glucose utilization. To this end, it is mandatory for stoichiometric models to incorporate energy use by action and synaptic potentials in addition to glutamatergic neurotransmission.

### Modulation of glucose uptake and cell-to-cell lactate shuttling by glutamate-glutamine cycle

The fate of carbons undergoing oxidative phosphorylation in neurons and astrocytes provides a quantitative hint of the relative amount of energy produced by aerobic pathways in the two cell types. We found that anaerobic and aerobic metabolism is similarly increased in both cell types at increasing activity (not shown). In particular, the fraction of cerebral oxidative metabolism in astrocytes is about 35% of the total, which is consistent with a substantial astrocytic contribution to functional brain energy metabolism (reviewed in [57]).

Analysis of glucose fluxes showed large fluctuations, so that both the neuron and the astrocyte may be the primary sites of glucose consumption at fixed values of Glu/Gln cycling. We indeed observed that both cell compartments can absorb from 10% to 90% of the total glucose uptake respectively. Therefore, at this level of detail, the network does not place significant constraints on the cellular utilization of glucose. The failure of up-regulation of ionic fluxes that we report here might play a role if their contribution is substantially different for neurons and astrocytes, which unfortunately has not been yet experimentally determined.

**Figure 6:**
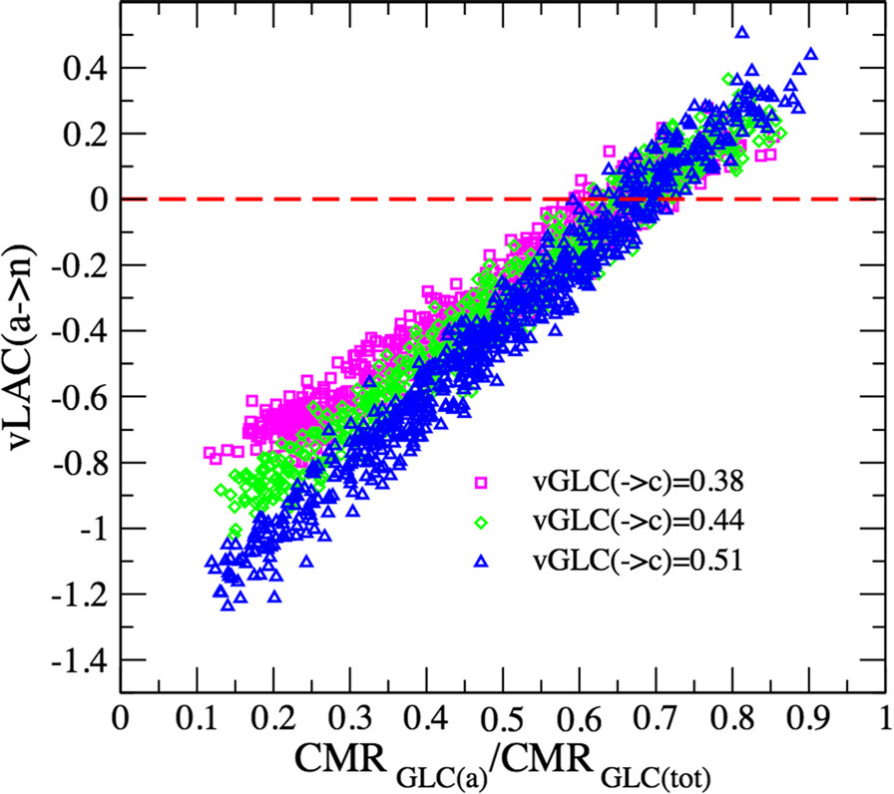
**Intercellular lactate flow versus glucose partitioning between neuron and astrocyte. **There is a clear dependence of the CCLS on the cell glucose uptake. However, when partitioning of glucose between neurons and astrocytes is around 65% (note that CMR_Glc(tot)_ = CMR_Glc(a)_+CMR_Glc(n)_), which also identifies the concomitant fraction of oxygen utilization, the contribution of transferred lactate is minimal. This means that he pyruvate derived from CCLS is thus always much less than the pyruvate generated by the concomitant uptake of glucose. Notably, if glucose is taken up equally by the neuronal and astrocytic compartments, the direction of lactate flow is preferentially from neurons to astrocytes (*ν*LAC(*a*→*n*) ≃ -0.3), contributing on average about 40% of the total carbons metabolized by these cells (note that carbons from lactate are obtained by considering the halved value of the flux).

The direction of the shuttle of lactate depends in a robust way on the sharing of glucose between neurons and astrocytes, resulting in ANLS when the relative astrocytic glucose uptake becomes larger than about 65% (implying that states supporting ANLS can be obtained by an *ad hoc* adjustment of Glc partitioning) and NALS otherwise (Figure 6). As the latter is also the mean value for the fraction of neuronal versus astrocytic oxygen utilization, it turns out that, on average, the contribution of CCLS to cell metabolism is very low compared to the lactate generated intracellularly by glucose. The strict dependence of CCLS on cellular glucose uptake supports previous results obtained through various modeling approaches [11,18,19].

**Figure 7:**
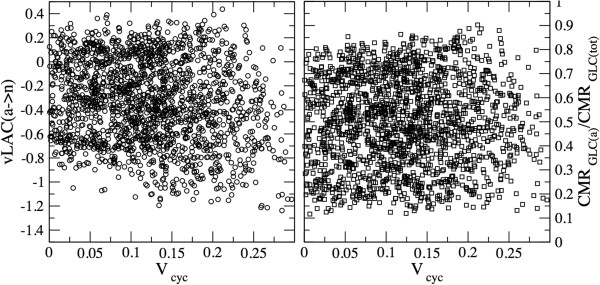
**Net ANLS flux (left) and relative cell glucose uptake (right) versus the velocity of the Glu/Gln cycle.** Independently of the correlation between *ν*LAC(*a* → *n*) and glucose partitioning observed in Figure 5, neither variable shows significant correlation with the activation level. This results from the absence of constraints imposed by stoichiometry on the rate of neurotransmission (hence on the glutamate-glutamine cycle), the latter being compatible with a large set of solutions relative to glucose partitioning and lactate shuttling.

A closer look at the individual solutions reveals the absence of a significant correlation between the glutamate–glutamine cycle and both the uptake of glucose and the shuttle of lactate (Figure 7). It should however be stressed that, as can be seen in Figure 7, the emerging scenario presents large fluctuations, in the sense that, even within the physiological range for the OGI, solutions with ANLS and NALS coexist. Together, these simulation outcomes indicate that no preferential route is undertaken by lactate at any given rate of glutamate-glutamine cycle. Thus, the determinants for lactate accumulation and shuttling, if any, must reside elsewhere, for example in the balance between spiking and synaptic activity [58].

### Conserved moieties, transcellular aspartate shuttling and glutathione-ascorbate cycle

To conclude, we discuss the flux organization of several pathways that were included in the metabolic network reconstruction, but whose involvement is not strictly related to lactate trafficking. The ATP buffering systems of creatine and adenylate kinase in neurons and astrocytes, as well as astrocytic glycogen metabolism were found to have negligible net flux, consistently with the role of adenylates, creatine and glycogen as conserved moieties in the steady-state. We are unable to test hypotheses about the role of brain glycogen [59-61], because our approach does not allow to describe the consumption of previously stored metabolites, such as glycogen in this case. We found that the contribution to NADH shuttling by the malate-aspartate shuttle (MAS) dominates over the glycerol-3-phosphate pathway (relative flux is a few percent). We could not support the recent hypothesis of a significant steady state neuron-to-astrocyte transport of aspartate [62]. However, this finding was expected due to the presence of mitochondrial aspartate-glutamate carrier in astrocytes, which allows for a shunting pathway, alternative to neuron-to-astrocyte aspartate transport that is sufficient to sustain NADH shuttling from cytosol to mitochondria in astrocytes, as previously suggested [28]. In order to examine a possible role of (dehydro)ascorbate transfer between neurons and astrocytes [63], we included the detoxification of reactive oxygen species (ROS) via glutathione/ascorbate cycle. Unluckily, we were unable to find statistically significant exchange fluxes of the two forms of ascorbic acids between cells. This is possibly correlated with the fact that, as said above, we did not include the entire network section associated with glutathione synthesis. Theoretical analysis of transcellular ascorbate cycling will thus require further refinements of the network reconstruction.

## Discussion

This work is concerned with a model of brain energy metabolism consisting of four compartments, representing neuron, astrocyte, extracellular space and the capillary. We used a steady state approach based on Von Neumann’s theoretical framework for the analysis of production networks [45], successfully applied to cellular metabolism in previous studies [21]. The steady-state assumption for the metabolic coupling between oxygen and glucose consumption underlying different cortical states is consistent with (i) the fact that experimental parameters have been measured under stationary conditions during suppressed brain activity [54], and (ii) the establishment of a new metabolic steady-state during enhanced brain activity [39]. At odds with standard schemes based on flux-balance analysis though, the frame employed here doesn’t constrain the net metabolite production to zero, but, rather, aims at recovering the steady state self-consistently from minimal stability requirements. The 'soft’ type of constraints thus arising makes it possible to sample the solution space corresponding to our large-scale network model in a statistically controlled manner, and returns a full range of feasible values for each reaction flux in the network, as well as detailed information on correlations. Based on this, one can elucidate the extent to which stoichiometry alone constrains the operation of brain metabolism, since the emerging picture is obtained without imposing specific functional constraints.

First, we found that the rate of glutamate-glutamine cycle distinguishes different activation states according to the fraction of glucose that is processed via glycolysis versus respiration. In particular, decreasing OGI values predict increasing velocity of the cycle, and viceversa. Notably, ATP-consuming Na ^+^ and K ^+^ fluxes across voltage- and ligand-gated ion channels are not directly dependent on glutamate-glutamine cycle in our theoretical account. Pathways analysis showed that their reaction rates do not “automatically” up-regulate along with transmitter cycling. This indicates that the absence of causal changes (i.e. constraints) in these different aspects of neuronal signaling strongly underestimates the glucose utilization at high activity levels. Unfortunately, it is presently unknown how to model the exact cause-and-effect relationship between glutamate-glutamine cycle and the ionic currents that generated neurotransmission on one hand, and those that are generated by neurotransmission on the other. The latter will constitute a primary target of future studies.

Second, the results of the present model support previous kinetic analyses indicating that lactate derived from astrocytes may not provide an important source of carbon compounds for neuronal energy metabolism in an activation-dependent manner (Figure 6) [11]. The model also supports the conclusion that the direction and magnitude of CCLS are secondary to glucose partitioning between cell types [11,19]. We found that glucose uptake, glycolysis and respiration change proportionally in a wide range of glutamate recycling rates. This implies that the glucose taken up by each cell is completely metabolized and not significantly converted to lactate. Thus, lactate does not accumulate in a specific cell type, i.e. the lactate concentration gradient, and hence lactate shuttle, remains small.

Third, simulations showed that glutamate-glutamine cycle is correlated with overall tissue glucose utilization and lactate production, but not with specific patterns of cellular glucose uptake and lactate shuttle (Figure 7). These results agree well with the experimental knowledge [54,64] and recover features of different (and sometimes conflicting) numerical studies performed previously [17,19]. Most importantly, they add further arguments to the idea that the trafficking of molecules between neurons and astrocytes underlies a broad functional, rather than strictly energetic partnership (see, e.g. [65]). This is evident in our modeling perspective, as possibly energy-related changes in lactate fluxes are independent of concomitant function-related variations in glutamate-glutamine cycle (see Figure 7, that essentially shows a lack of correlation between lactate shuttling and transmitter cycling). Alternative functions for lactate include the discrimination between dilation and constriction of cerebral arterioles [66], or the modulation of GABAergic inhibitory activity of specific neuronal populations [67]. These functional roles of lactate might be still secondary to its accumulation in the tissue. Our simulations suggest that this accumulation likely results from up-regulation of non-oxidative metabolism in both neurons and astrocytes, as previously suggested [11,60].

We found that intercellular shuttling of lactate increases only when glucose partitioning between neurons and astrocytes is significantly uneven (Figure 6). Therefore, lactate transfer can be interpreted as a local biochemical shunt that allows for the optimal use of carbon supply in correspondence of variable environmental challenges.

Interestingly, if the functional partnership between neurons and astrocytes is suppressed via the zeroing of neurotransmission, the anticorrelation between glycolysis in the two cellular compartments further increases (i.e. correlation becomes more negative), a feature shared with many other fluxes related to energetics, including TCA cycle and oxidative phosphorylation. So, even when there is no functional relationship between neurons and astrocytes, their energy (primarily glycolytic) metabolism is anticorrelated. The reason for this behavior is that the two cell types share the same environment, thereby the same glucose availability. Glucose availability thus represents the primary drive on glucose partitioning. If the two cells are functionally independent, oxidative metabolism follows glycolysis, as also evidenced by the negative correlation between neuronal and astrocytic oxidative metabolism. As soon as neurons and astrocytes become coupled by neurotransmission, the network shifts to correlated patterns of activity, both within the same compartment and between different compartments. Accordingly, glycolysis in one cell type becomes slightly positively correlated with oxidative metabolism in the other. However, glycolysis in either neurons or astrocytes, which essentially reflect partitioning of tissue glucose, remains poorly correlated with the glutamate-glutamine cycle. Overall, glutamate-glutamine cycle positively correlates with cell TCA cycle and oxidative phosphorylation much more than it does with glycolysis, which is especially significant for the astrocytic compartment.

## Conclusions

In conclusion, we developed a large-scale model for compartmentalized brain energy metabolism including the core carbon pathways of neurons and astrocytes, as well as further compartments (capillary, extracellular space). A constraint-based scheme was then employed in order to define feasible configurations of reaction fluxes. Our numerical analysis was based on a relaxation algorithm which allows to obtain a statistically controlled sampling of the solution space without any prior assumption on the behaviour of energy producing/consuming pathways. Results have shown that only a large imbalance of cell glucose uptake can explain the occurrence of a significant lactate shuttle between neurons and astrocytes, the latter being roughly independent of the rates of transmitter cycling. Our results therefore do not support a link between glutamate-glutamine cycle and CCLS as a mechanism for activation-dependent transfer of energy compounds within the brain. On the other hand, CCLS can be found by assuming that neurons have limited access to glucose and/or by bounding their glucose uptake flux. The lack of correlation we observe stems from the fact that the distribution of activity-related energy stress between neurons and astrocytes cannot be estimated by the stoichiometry of the metabolic network and should be a primary target of current experimental research. Future developments will focus on introducing minimal *ad hoc* constraints on neurotransmitter cycling and ionic fluxes in the hope to capture the non-stoichiometric side of the energetics of brain activity.

## Endnote

^a^ Because of finite numerical precision, for our putposes a flux is null when it is smaller than 10^-6^.

## Competing interests

The authors declare that they have no competing interests.

## Authors’ contributions

ADM, MDN, FG and EM designed research; all authors contributed data and/or analysis methods; FAM performed research, with contributions from all authors; all authors analyzed results; all authors drafted the manuscript. All authors read and approved the final manuscript.

## Supplementary Material

Additional file 1 Supporting text. Click here for file
