# Supplementary material for: Energy metabolism and glutamate-glutamine cycle in the brain: a stoichiometric modeling perspective
Source: BMC Syst Biol. 2013 Oct 10;7:103. doi: 10.1186/1752-0509-7-103 (PMC4021976; doi:10.1186/1752-0509-7-103)
Supplement: Additional file 1 — Supporting text. [file 1752-0509-7-103-S1.pdf]

SUPPORTING TEXT : Energy metabolism and glutamate-glutamine cycle in the brain: A stoichiometric modeling perspective

Francesco A. Massucci[1], Mauro DiNuzzo[2,3], Federico Giove[2,3], Bruno Maraviglia[3,4], Isaac Perez Castillo[5], Enzo Marinari[3,6,‡] and Andrea De Martino[*3,6,7,‡]

[1]Departament d'Enginyeria Quimica, Universitat Rovira i Virgili, 43007 Tarragona (Spain)
[2]Magnetic Resonance for Brain Investigation Lab, Enrico Fermi Center, Roma (Italy)
[3]Dipartimento di Fisica, Sapienza Università di Roma, P.le Aldo Moro 2, 00185 Roma (Italy)
[4]Fondazione Santa Lucia, Roma (Italy)
[5]Department of Mathematics, King's College London, Strand, London, WC2R 2LS (UK)
[6]Center for Life Nano Science@Sapienza, Istituto Italiano di Tecnologia, Viale Regina Elena 291, 00161 Roma (Italy)
[7]CNR–IPCF, Unità di Roma Sapienza, Roma (Italy)
[‡]These authors contributed equally to this work

Email: andrea.demartino@roma1.infn.it;

[*]Corresponding author

1 Constraint-based flux model

The standard approach to modeling biochemical reaction networks is based on enzyme kinetics and is normally formulated through systems of differential equations for the time evolution of the intracellular concentrations of metabolites, like

$$\dot{\mathbf{c}} = \mathbf{S}\boldsymbol{\nu} - \mathbf{u} \tag{1}$$

where $\mathbf{c}$ denotes the concentration vector, $\mathbf{S}$ the matrix of stoichiometric indices, $\mathbf{v}$ the flux vector (in turn, a function of concentrations, kinetic constants $\mathbf{k}$, etc.: $\boldsymbol{\nu} \equiv \boldsymbol{\nu}(\mathbf{c}, \mathbf{k}, \ldots)$), and $\mathbf{u}$ the vector of uptakes describing the exchange of chemical species with the environment. Limited knowledge of enzyme mechanisms and kinetic constants as well as the fast increase in the number of parameters, however, effectively circumscribes this framework's applicability to small systems (up to a few tens of reactions as in the case of human erythrocites [1]). Large- (possibly genome-) scale reaction networks require, to date, a different, simplified type of analysis.

Constraint based models are usually defined by the requirement that in non equilibrium steady states (NESS) the net rate of change of the level of metabolites is zero (i.e. by homeostasis). As a consequence,

(1) reduces to

$$\mathbf{S}\boldsymbol{\nu} = \mathbf{u} \quad , \tag{2}$$

and one is interested in retrieving the flux patterns $\mathbf{v}$ that are consistent with a NESS induced by the (given) vector $\mathbf{u}$ of boundary fluxes [2–8]. In essence, constraint-based models retain the information encoded in $\mathbf{S}$ as the key input and use it to define, through (2), a polytope of dimension $D = N - \text{rank}(\mathbf{S})$ (N being the number of reactions in the network) that, if complemented with physiological bounds on the flux variables and with detailed prescriptions for in- and out-takes, can provide a sensible representation of the metabolic capabilities of the cell in a given medium. In addition, they allow for the straightforward integration of biochemical, empirical or thermodynamic data (when available) into the problem, either as specific bounds on fluxes (e.g. for reaction directionality) or in the form of extra constraints (e.g. conservation laws). In the present study, each flux ν_i ($i = 1, \ldots, N$) is assumed to be either irreversible, in which case its bound of variability is simply $0 \leq \nu_i < \infty$, or reversible, in which case $-\infty < \nu_i < \infty$. In the latter case, however, we introduce two irreversible fluxes to describe the forward and reverse processes respectively, so that, once every reversible reaction is split in two, all bounds we consider are of the form $0 \leq \nu_i < \infty$ (except for the glucose uptake flux to the capillary which, as explained in the main text, is assumed to be fixed.)

For many microbial metabolic networks $\mathbf{S}$ is known with gene-level accuracy. In such cases, a characterization of the cell's metabolism can be obtained by sampling (ideally with uniform probability) the space of feasible network configurations defined by (2). Unluckily, the task of generating solutions uniformly out of the polytope is computationally unaffordable when D becomes larger than a few tens (for typical genome-scale networks, D can be as large as several hundreds) [9, 10]. For systems like bacteria, however, it is possible to reduce the complexity of the solution space by coupling (2) with the optimization of a (usually linear) score function representing the biological functionality of the organism in the selected extracellular conditions (e.g. biomass flux maximization under optimal growth conditions for E. coli) [11–14]. This type of approach provides a further unquestionable advantage in terms of computational tractability and genome-scale models of metabolism have been developed along these lines, for several single-cell organisms.

For cells carrying no clear objective function the latter approach is much harder to justify and sampling solutions appears as the most logical step to take. A viable alternative to (2) in such cases consists in relaxing the mass-balance constraint to allow for a net production of chemical species, while leaving the network functionally unconstrained so that the metabolite production profile can be determined self-consistently.

This scenario is described by the system of inequalities

$$\mathbf{S}\boldsymbol{\nu} \geq \mathbf{0} \quad , \tag{3}$$

which are easily obtained from (2) if one includes in- and out-takes in $\mathbf{S}$ (and the corresponding fluxes in $\mathbf{v}$). Steady-state conditions like (3) were originally introduced by Von Neumann in the analysis of input-output networks [15] and simply state that, in non equilibrium steady states, flux configurations in which the network produces a metabolite in excess of consumption are allowed. In particular, solving (3) for $\boldsymbol{\nu}$ after fixing a configuration of in-takes (out-takes being in this case an outcome) allows to retrieve an M-dimensional vector $\mathbf{y} = \mathbf{S}\boldsymbol{\nu}$ whose entries encode information on whether metabolite j is being produced ($y_j > 0$) or not ($y_j = 0$) in that particular solution [16,17].

The physiological rationale to employ such constraints is two-fold. On one hand, a net production of certain chemical species (e.g. amino acids) must be expected to take place if macromolecular processes outside metabolism strictly defined (e.g. proteinogenesis) are to occur. In absence of an objective function that accounts for such processes, (3) appear as a reasonable minimal constraints for the metabolic capabilities of a cell, in the sense that they don't even impose which chemical species are to be globally produced. On the other hand, when the network reconstruction is incomplete one should complement (2) with additional constraints that account for the flow of chemical species to network modules not included in the model. Such out-takes may be hard to implement in absence of detailed genomic data. Applying (3) to partial network reconstructions allows to deal with this issue rather naturally, by exploring all possible metabolic exchanges compatible with the given stoichiometry. Moreover, its dual problem has recently been given a thermodynamic interpretation in the context of cell metabolism [18,19].

2 Numerical analysis: relaxation method

The flux problem (3) has been studied from a purely theoretical perspective in [20–23]. The technical advantage that accompanies (3) lies in the existence of a computationally efficient, statistically controlled method to generate solutions. The procedure consists in essence of a Relaxation algorithm [24] based on the analogy of (3) with perceptron learning [25] and is described in detail in [21]. In brief, denoting by $\mathbf{A}$ and $\mathbf{B}$ the matrices of input and output stoichiometric indices respectively (so that $\mathbf{S} = \mathbf{B} - \mathbf{A}$), let $\rho > 0$ be a real parameter, let $\mathbf{S}_\rho = \mathbf{B} - \rho\mathbf{A}$, and consider the system

$$\mathbf{S}_\rho\boldsymbol{\nu} \geq \mathbf{0} \quad . \tag{4}$$

Given a (generic) flux vector $\boldsymbol{\nu}$ (e.g. randomly generated from a prescribed probability distribution), a solution of (4) can be found for any fixed $\rho < 1$ by the following algorithm:

- compute $\mathbf{y} = \mathbf{S}_\rho \boldsymbol{\nu}$ and $j_0 = \arg\min_j y_j$ (i.e., j_0 is the index of the least satisfied constraint);
- if $y_{j_0} \geq 0$ then $\boldsymbol{\nu}$ is a solution of (4); exit.
- if $y_{j_0} < 0$ then update $\boldsymbol{\nu}$ component-wise as

$$\boldsymbol{\nu} \;\to\; \max\{\mathbf{0}, \boldsymbol{\nu} + \lambda \mathbf{S}_\rho^{(j_0)}\} \tag{5}$$

  where $\lambda > 0$ is a constant and $\mathbf{S}_\rho^{(k)}$ is the k-th row of matrix $\mathbf{S}_\rho$; go to 2 and iterate.

This is a classical relaxation procedure. Intuitively, a violated constraint ($y_j < 0$) signals that the consumption of metabolite j exceeds its production. The flux of reactions where j participates is then modified so as to increase the production flux and decrease the consumption flux. This procedure converges to a solution for each $\rho < 1$ [21] so that the solutions in the limit $\rho \to 1$, where the original problem is recovered, can be obtained by increasing ρ recursively to approach 1 with the desired precision and extrapolating. Notice that the form of the update step (5) guarantees that the bounds of variability $0 \leq \nu_i < \infty$ are satisfied.

Disposing of large sets of solutions allows to evaluate many quantities of interest like the statistics of production profiles, marginal flux distributions, flux-flux correlations and, of course, distributions of "macroscopic" observables like, in our case, the OGI or the CMR (which are defined through simple functions of the individual fluxes). This approach has been applied in different contexts to quantify the metabolic capabilities of cells [26–28]. Following the above procedure, different solutions can be generated by re-initializing the algorithm from different flux vectors. A crucial question concerns the statistics of the solution space sampling that can thus be obtained. This problem has been faced in [19]. In brief, if one fixes the probability distribution from which initial conditions are generated ('priors' for short), repeated iteration of the above scheme provides a set of solutions that minimize the *average* Euclidean distance between the solutions and the priors. More precisely, let us assume that (for each ρ) initial conditions are drawn from a fixed, 'trial' probability distribution $P_0(\boldsymbol{\nu})$ of flux vectors (for simplicity, one may think that $P_0(\boldsymbol{\nu}) = \prod_{i=1}^N P_0^{(i)}(\nu_i)$, with prescribed distributions $P_0^{(i)}$, e.g. uniform over a given interval: in this case each initial v_i is selected randomly and independently from its trial distribution $P_0^{(i)}$). Then the solutions $\boldsymbol{\nu}^\star$ obtained by the above method are such that the quantity

$$d^2 = \left\langle \sum_{i=1}^N (\nu_i^\star - \nu_i)^2 \right\rangle \tag{6}$$

is minimized (the average being taken over P_0). In other words, one obtains a set of solutions that are as close as possible to the priors used to generate them and the statistical significance of the corresponding distributions of fluxes (or other relevant macroscopic observables like the OGI etc.) can be interpreted in this light. In essence, multiple (random) initializations of the above algorithm deform the uncorrelated trial distributions $P_0^{(i)}$ to generate a set of correlated probability distributions for the ν_i's, the correlation being driven by the form of the reinforcement term. Note that, quite importantly, the resulting ν_i's can exceed the initial bounds defined by P_0.

Now it is clear that the solution space picture one obtains can depend strongly on the choice of the priors. On one hand, disposing of sufficient empirical information about individual fluxes one can inject it into the prior (i.e. into $P_0^{(i)}$, e.g. by assuming that such distributions are uniform and centered around the empirical value) to evaluate the extent to which the solution space is constrained by sampling configurations "close" (on average) to such a prior. However, in the case we consider, such an information is not available in the amount and precision that would be needed. In situations like these it is reasonable to think that priors should inject into the problem as little information as possible so as to obtain unbiased information on the solution space (besides the global constraint imposed by te minimization of (6)). This approach has the advantage of providing a portrait of the solution space that is minimally constrained by the prior, in the sense that the emergent features are not due to the external biochemical information employed but, strictly speaking, to the topology of the network and the functional constraints (in our case embodied only by the OGI). In this work we have followed such a prescription.

In summary, we have solved (3) using the above method for the (partial) network reconstruction described in following section. For the trial functions $P_0^{(i)}$, we took uniform distributions on $[0, 1]$ (as said above, the initial bounds of variability allowed for the fluxes can be exceeded by the algorithm). The only additional constraint imposed on the solution space of (3) is given by the uptake of GLC to the capillary, which is fixed and tuned externally. All other fluxes are computed self-consistently. This implies that our solution space may contain unphysiologic states that we do not discount a priori. Our focus is indeed on exploring a minimally constrained solution space. Assessing the robustness of the emerging scenario against physiological data instead highlights which bounds on variables/constraints should *necessarily* be added to non-equilibrium steady state models in order to reproduce a realistic phenomenology.

3 Network reconstruction: details

We reconstruct the cerebral metabolism via a compartmentalized model divided into blood capillary (c), extracellular space (e), neuron (n) and astrocyte (a). The last two compartments are then divided into cytosol (nc and ac respectively) and mitochondria (nm and am), while we also consider vesicles (nv) in synapses, from where the neurotransmitter glutamate is released to the extracellular medium. Considering the reversibility of reactions the network altogether encompasses 139 chemical reactions, reported in table 1, which process 108 chemical species, listed in table 2. Reaction names are determined by the catalyzing enzymes or, in the case of transport processes, by the chemical compound itself. The compartments involved by the chemical processes are also specified, so that, for example, $\nu\text{HK}(n)$ denotes the reaction catalyzed by the hexokinase in neurons, while $\nu\text{O}_2(c \to n)$ indicates an oxygen transport from the capillary to the neuron. In case of reversible reactions, the forward and reverse processes are separated into two different reactions, labeled with f and r respectively.

Since we are mainly interested in the carbohydrate metabolism and related energetic production, we specifically consider pathways involved in those processes plus some other features peculiar of cerebral cells, like for instance creatine synthesis and metabolism. Apart from oxygen, lactate and glucose transport from/to the capillary vessel and within the system, we have considered 14 main paths, which allow the system to self-sustain itself. We included glycolysis, pentose phosphate pathway, Tricarboxylic acid cycle (TCA), oxidative phosphorylation, which are all inherent to carbohydrate metabolism. To allow detoxification of species produced by the latter we then included the Glutathione-ascorbate (GSH/ASC) cycle, while transport of reducing equivalents (NADH) is performed through the Malate Aspartate shuttle, in turn coupled with the pentose phosphate pathway and the NAD/NADP interconversion. The coupling between functionality and metabolism is assured through neurotransmission and transmitter recycling, which together with the ionic movements provides a metabolic interconnection between neurons and astrocytes.

Table 1: List of reactions.

	Abbreviation	*Chemical Reaction*

GLC, O_2 and LAC Transport

1	νGLC(c)	$\rightarrow GLC_c$
2	νGLC($c \rightarrow a$)	$GLC_c \rightarrow GLC_a$
3	νGLC($c \rightarrow e$)	$GLC_c \rightarrow GLC_e$
4	νGLC($e \rightarrow a$)	$GLC_e \rightarrow GLC_a$
5	νGLC($e \rightarrow n$)	$GLC_e \rightarrow GLC_n$
6	$\nu O_2(c)$	$\rightarrow O_{2c}$
7	$\nu O_2(c \rightarrow a)$	$O_{2c} \rightarrow O_{2a}$
8	$\nu O_2(c \rightarrow n)$	$O_{2c} \rightarrow O_{2n}$
9	νLAC(c)	$LAC_c \rightarrow$
10	νLAC($e \rightarrow c$)	$LAC_e \rightarrow LAC_c$
11	νLAC($a \rightarrow c$)	$LAC_a \rightarrow LAC_c$
12	νLAC($e \rightarrow a$)	$LAC_e \rightarrow LAC_a$
13	νLAC($a \rightarrow e$)	$LAC_a \rightarrow LAC_e$
14	νLAC($e \rightarrow n$)	$LAC_e \rightarrow LAC_n$
15	νLAC($n \rightarrow e$)	$LAC_n \rightarrow LAC_e$

Creatine & Adenylate Kinase Buffers

16	$\nu CK^f(n)$	$ATP_n + Cr_n \rightarrow ADP_n + PCr_n$
17	$\nu CK^r(n)$	$ADP_n + PCr_n \rightarrow ATP_n + Cr_n$
18	$\nu CK^f(a)$	$ATP_a + Cr_a \rightarrow ADP_a + PCr_a$
19	$\nu CK^r(a)$	$ADP_a + PCr_a \rightarrow ATP_a + Cr_a$
20	$\nu AK^f(n)$	$2\ ADP_n \rightarrow AMP_n + ATP_n$
21	$\nu AK^r(n)$	$AMP_n + ATP_n \rightarrow 2\ ADP_n$
22	$\nu AK^f(a)$	$2\ ADP_a \rightarrow AMP_a + ATP_a$
23	$\nu AK^r(a)$	$AMP_a + ATP_a \rightarrow 2\ ADP_a$

Pentose Phosphate Pathway

24	νPPP(n)	$3\ G6P_n + 6\ NADP_n \rightarrow 2\ F6P_n + GAP_n + 6\ NADPH_n$
25	νPPP(a)	$3\ G6P_a + 6\ NADP_a \rightarrow 2\ F6P_a + GAP_a + 6\ NADPH_a$

Ionic movements

26	νNAK(n)	$ATP_n + 2\ K_e + 3\ Na_n \rightarrow ADP_n + 2\ K_n + 3\ Na_e$
27	νNAK(a)	$ATP_a + 2\ K_e + 3\ Na_a \rightarrow ADP_a + 2\ K_a + 3\ Na_e$
28	νNKCC(a)	$K_e + Na_e \rightarrow K_a + Na_a$
29	νNa(n)	$Na_e \rightarrow Na_n$
30	νK(n)	$K_n \rightarrow K_e$

Table 1: List of reactions.

	Abbreviation	*Chemical Reaction*

GSH/ASC Cycle (metabolism and transport)

31	$\nu\mathrm{GR}^1(\mathrm{n})$	$\mathrm{GSSG_n + NADPH_n \to GSH_n + NADP_n}$
32	$\nu\mathrm{GR}^2(n)$	$\mathrm{GSSG_n + NADPH_{nm} \to GSH_n + NADP_{nm}}$
33	$\nu\mathrm{DHAR}(n)$	$\mathrm{DHA_n + GSH_n \to ASC_n + GSSG_n}$
34	$\nu\mathrm{APX}(n)$	$\mathrm{ASC_n + ROS_n \to DHA_n}$
35	$\nu\mathrm{GR}^1(a)$	$\mathrm{GSSG_a + NADPH_a \to GSH_a + NADP_a}$
36	$\nu\mathrm{GR}^2(a)$	$\mathrm{GSSG_a + NADPH_{am} \to GSH_a + NADP_{am}}$
37	$\nu\mathrm{DHAR}(a)$	$\mathrm{DHA_a + GSH_a \to ASC_a + GSSG_a}$
38	$\nu\mathrm{APX}(a)$	$\mathrm{ASC_a + ROS_a \to DHA_a}$
39	$\nu\mathrm{GSH}(a)$	$\to \mathrm{GSH_a}$
40	$\nu\mathrm{GSH}(a \to e)$	$\mathrm{GSH_a \to GSH_e}$
41	$\nu\mathrm{GSH}(e \to n)$	$\mathrm{GSH_e \to GSH_n}$
42	$\nu\mathrm{DHA}(n \to e)$	$\mathrm{DHA_n \to DHA_e}$
43	$\nu\mathrm{DHA}(e \to n)$	$\mathrm{DHA_e \to DHA_n}$
44	$\nu\mathrm{DHA}(e \to a)$	$\mathrm{DHA_e \to DHA_a}$
45	$\nu\mathrm{DHA}(a \to e)$	$\mathrm{DHA_a \to DHA_e}$
46	$\nu\mathrm{ASC}(a \to e)$	$\mathrm{ASC_a \to ASC_e}$
47	$\nu\mathrm{ASC}(e \to a)$	$\mathrm{ASC_e \to ASC_a}$
48	$\nu\mathrm{ASC}(e \to n)$	$\mathrm{ASC_e + 2\ Na_e \to ASC_n + 2\ Na_n}$

Neurotransmission and GLU/GLN Cycle

49	$\nu\mathrm{GLU}(e \to a)$	$\mathrm{GLU_e + K_a + 3\ Na_e \to GLU_{ac} + K_e + 3\ Na_a}$
50	$\nu\mathrm{GS}(a)$	$\mathrm{ATP_a + GLU_{ac} \to ADP_a + GLN_a}$
51	$\nu\mathrm{GLN}(a \to n)$	$\mathrm{GLN_a \to GLN_n}$
52	$\nu\mathrm{PAG}(n)$	$\mathrm{GLN_n \to GLU_{nc}}$
53	$\nu\mathrm{GLU}(n)$	$\mathrm{ATP_n + GLU_{nc} \to ADP_n + GLUnv}$
54	$\nu\mathrm{NT}(n \to e)$	$\mathrm{GLUnv \to GLU_e}$

Table 1: List of reactions.

	Abbreviation	*Chemical Reaction*

Glycolysis and Glycogenolysis

55	νHK(n)	$ATP_n + GLC_n \rightarrow ADP_n + G6P_n$
56	νPFK(n)	$ATP_n + G6P_n \rightarrow ADP_n + 2\ GAP_n$
57	νGAPDH^f(n)	$GAP_n + NAD_{nc} \rightarrow BPG_n + NADH_{nc}$
58	νGAPDH^r(n)	$BPG_n + NADH_{nc} \rightarrow GAP_n + NAD_{nc}$
59	νPGK^f(n)	$ADP_n + BPG_n \rightarrow ATP_n + PEP_n$
60	νPGK^r(n)	$ATP_n + PEP_n \rightarrow ADP_n + BPG_n$
61	νPK(n)	$ADP_n + PEP_n \rightarrow ATP_n + PYR_{nc}$
62	νLDH^f(n)	$NADH_{nc} + PYR_{nc} \rightarrow LAC_n + NAD_{nc}$
63	νLDH^r(n)	$LAC_n + NAD_{nc} \rightarrow NADH_{nc} + PYR_{nc}$
64	νHK(a)	$ATP_a + GLC_a \rightarrow ADP_a + G6P_a$
65	νPFK(a)	$ATP_a + G6P_a \rightarrow ADP_a + 2\ GAP_a$
66	νGAPDH^f(a)	$GAP_a + NAD_{ac} \rightarrow BPG_a + NADH_{ac}$
67	νGAPDH^r(a)	$BPG_a + NADH_{ac} \rightarrow GAP_a + NAD_{ac}$
68	νPGK^f(a)	$ADP_a + BPG_a \rightarrow ATP_a + PEP_a$
69	νPGK^r(a)	$ATP_a + PEP_a \rightarrow ADP_a + BPG_a$
70	νPK(a)	$ADP_a + PEP_a \rightarrow ATP_a + PYR_{ac}$
71	νLDH^f(a)	$NADH_{ac} + PYR_{ac} \rightarrow LAC_a + NAD_{ac}$
72	νLDH^r(a)	$LAC_a + NAD_{ac} \rightarrow NADH_{ac} + PYR_{ac}$

Pyruvate Shuttling to Mitochondria

73	νPYR^f(n)	$PYR_{nc} \rightarrow PYR_{nm}$
74	νPYR^r(n)	$PYR_{nm} \rightarrow PYR_{nc}$
75	νPYR^f(a)	$PYR_{ac} \rightarrow PYR_{am}$
76	νPYR^r(a)	$PYR_{am} \rightarrow PYR_{ac}$

Oxidative Phosphorylation

77	νOP(n)	$5\ ADP_n + 2\ NADH_{nm} + O_{2n} \rightarrow 5\ ATP_n + 2\ NAD_{nm} + 0.01\ ROS_n$
78	νOP(a)	$5\ ADP_a + 2\ NADH_{am} + O_{2a} \rightarrow 5\ ATP_a + 2\ NAD_{am} + 0.01\ ROS_a$

NAD/NADP Interconversion

79	νNAD(n)	$NADH_{nm} + NADP_{nm} \rightarrow NADPH_{nm} + NAD_{nm}$
80	νNAD(a)	$NADH_{am} + NADP_{am} \rightarrow NADPH_{am} + NAD_{am}$

Anaplerosys

81	νPC(a)	$ATP_a + PYR_{am} \rightarrow ADP_a + OAA_{am}$
82	νcME(n)	$MAL_{nc} + NADP_n \rightarrow NADPH_{nc} + PYR_{nc}$
83	νmME(n)	$MAL_{nm} + NADP_{nm} \rightarrow NADPH_{nm} + PYR_{nm}$
84	νcME(a)	$MAL_{ac} + NADP_a \rightarrow NADPH_{ac} + PYR_{ac}$
85	νmME(a)	$MAL_{am} + NADP_{am} \rightarrow NADPH_{am} + PYR_{am}$

Table 1: List of reactions.

	Abbreviation	*Chemical Reaction*
	**TCA Cycle**	
86	νPDH(n)	$CoA_n + NAD_{nm} + PYR_{nm} \rightarrow ACoA_n + NADH_{nm}$
87	νCS(n)	$ACoA_n + OAA_{nm} \rightarrow CIT_n + CoA_n$
88	$\nu IDH^1(n)$	$CIT_n + NAD_{nm} \rightarrow AKG_{nm} + NADH_{nm}$
89	$\nu IDH^2(n)$	$CIT_n + NADP_{nm} \rightarrow AKG_{nm} + NADPH_{nm}$
90	$\nu IDH^3(n)$	$CIT_n + NADP_n \rightarrow AKG_{nc} + NADPH_n$
91	νAKGDH(n)	$AKG_{nm} + CoA_n + NAD_{nm} \rightarrow NADH_{nm} + SCoA_n$
92	$\nu SCoATK^f(n)$	$ADP_n + SCoA_n \rightarrow ATP_n + SUC_n$
93	$\nu SCoATK^r(n)$	$ATP_n + SUC_n \rightarrow ADP_n + SCoA_n$
94	$\nu SDH^f(n)$	$1.50\ ADP_n + 0.10\ O_{2n} + 3\ SUC_n \rightarrow 1.50\ ATP_n + 3\ FUM_n$
95	$\nu SDH^r(n)$	$1.50\ ATP_n + 3\ FUM_n \rightarrow 1.50\ ADP_n + 0.10\ O_{2n} + 3\ SUC_n$
96	$\nu FUM^f(n)$	$FUM_n \rightarrow MAL_{nm}$
97	$\nu FUM^r(n)$	$MAL_{nm} \rightarrow FUM_n$
98	νmMDH(n)	$MAL_{nm} + NAD_{nm} \rightarrow NADH_{nm} + OAA_{nm}$
99	νPDH(a)	$CoA_a + NAD_{am} + PYR_{am} \rightarrow ACoA_a + NADH_{am}$
100	νCS(a)	$ACoA_a + OAA_{am} \rightarrow CIT_a + CoA_a$
101	$\nu IDH^1(a)$	$CIT_a + NAD_{am} \rightarrow AKG_{am} + NADH_{am}$
102	$\nu IDH^2(a)$	$CIT_a + NADP_{am} \rightarrow AKG_{am} + NADPH_{am}$
103	$\nu IDH^3(a)$	$CIT_a + NADP_a \rightarrow AKG_{ac} + NADPH_a$
104	νAKGDH(a)	$AKG_{am} + CoA_a + NAD_{am} \rightarrow NADH_{am} + SCoA_a$
105	$\nu SCoATK^f(a)$	$ADP_a + SCoA_a \rightarrow ATP_a + SUC_a$
106	$\nu SCoATK^r(a)$	$ATP_a + SUC_a \rightarrow ADP_a + SCoA_a$
107	$\nu SDH^f(a)$	$1.50\ ADP_a + 0.10\ O_{2a} + 3\ SUC_a \rightarrow 1.50\ ATP_a + 3\ FUM_a$
108	$\nu SDH^r(a)$	$1.50\ ATP_a + 3\ FUM_a \rightarrow 1.50\ ADP_a + 0.10\ O_{2a} + 3\ SUC_a$
109	$\nu FUM^f(a)$	$FUM_a \rightarrow MAL_{am}$
110	$\nu FUM^r(a)$	$MAL_{am} \rightarrow FUM_a$
111	νmMDH(a)	$MAL_{am} + NAD_{am} \rightarrow NADH_{am} + OAA_{am}$
	**Glycerol 3-P Shuttle**	
112	νG3PS(n)	$1.50\ ADP_n + 3\ NADH_{nc} + 0.10\ O_{2n} \rightarrow 1.50\ ATP_n + 3\ NAD_{nc}$
113	νG3PS(a)	$1.50\ ADP_a + 3\ NADH_{ac} + 0.10\ O_{2a} \rightarrow 1.50\ ATP_a + 3\ NAD_{ac}$
	**MAS**	
114	νcMDH(n)	$NADH_{nc} + OAA_{nc} \rightarrow MAL_{nc} + NAD_{nc}$
115	νOGC(n)	$AKG_{nm} + MAL_{nc} \rightarrow AKG_{nc} + MAL_{nm}$
116	νAGC(n)	$ASP_{nm} + GLU_{nc} \rightarrow ASP_{nc} + GLU_{nm}$
117	νcMDH(a)	$NADH_{ac} + OAA_{ac} \rightarrow MAL_{ac} + NAD_{ac}$
118	νOGC(a)	$AKG_{am} + MAL_{ac} \rightarrow AKG_{ac} + MAL_{am}$
119	νAGC(a)	$ASP_{am} + GLU_{ac} \rightarrow ASP_{ac} + GLU_{am}$

Table 1: List of reactions.

Abbreviation	*Chemical Reaction*

Aspartate Metabolism and Shuttling

120 $\nu\mathrm{cAAT}^{\mathrm{f}}(n)$	$\mathrm{AKG_{nc}} + \mathrm{ASP_{nc}} \rightarrow \mathrm{GLU_{nc}} + \mathrm{OAA_{nc}}$
121 $\nu\mathrm{cAAT}^{\mathrm{r}}(n)$	$\mathrm{GLU_{nc}} + \mathrm{OAA_{nc}} \rightarrow \mathrm{AKG_{nc}} + \mathrm{ASP_{nc}}$
122 $\nu\mathrm{mAAT}^{\mathrm{f}}(n)$	$\mathrm{AKG_{nm}} + \mathrm{ASP_{nm}} \rightarrow \mathrm{GLU_{nm}} + \mathrm{OAA_{nm}}$
123 $\nu\mathrm{mAAT}^{\mathrm{r}}(n)$	$\mathrm{GLU_{nm}} + \mathrm{OAA_{nm}} \rightarrow \mathrm{AKG_{nm}} + \mathrm{ASP_{nm}}$
124 $\nu\mathrm{cAAT}^{\mathrm{f}}(a)$	$\mathrm{AKG_{ac}} + \mathrm{ASP_{ac}} \rightarrow \mathrm{GLU_{ac}} + \mathrm{OAA_{ac}}$
125 $\nu\mathrm{cAAT}^{\mathrm{r}}(a)$	$\mathrm{GLU_{ac}} + \mathrm{OAA_{ac}} \rightarrow \mathrm{AKG_{ac}} + \mathrm{ASP_{ac}}$
126 $\nu\mathrm{mAAT}^{\mathrm{f}}(a)$	$\mathrm{AKG_{am}} + \mathrm{ASP_{am}} \rightarrow \mathrm{GLU_{am}} + \mathrm{OAA_{am}}$
127 $\nu\mathrm{mAAT}^{\mathrm{r}}(a)$	$\mathrm{GLU_{am}} + \mathrm{OAA_{am}} \rightarrow \mathrm{AKG_{am}} + \mathrm{ASP_{am}}$
128 $\nu\mathrm{ASP}^{\mathrm{f}}(n \rightarrow a)$	$\mathrm{ASP_{nc}} \rightarrow \mathrm{ASP_{ac}}$
129 $\nu\mathrm{ASP}^{\mathrm{r}}(n \rightarrow a)$	$\mathrm{ASP_{ac}} \rightarrow \mathrm{ASP_{nc}}$
130 $\nu\mathrm{ASP}^{\mathrm{f}}(n)$	$\mathrm{ASP_{nc}} \rightarrow \mathrm{ASP_{nm}}$
131 $\nu\mathrm{ASP}^{\mathrm{r}}(n)$	$\mathrm{ASP_{nm}} \rightarrow \mathrm{ASP_{nc}}$
132 $\nu\mathrm{ASP}^{\mathrm{f}}(a)$	$\mathrm{ASP_{ac}} \rightarrow \mathrm{ASP_{am}}$
133 $\nu\mathrm{ASP}^{\mathrm{r}}(a)$	$\mathrm{ASP_{am}} \rightarrow \mathrm{ASP_{ac}}$

Glutamate Metabolism

134 $\nu\mathrm{GDH}^{\mathrm{f}}(n)$	$\mathrm{GLU_{nm}} + \mathrm{NADP_{nm}} \rightarrow \mathrm{AKG_{nm}} + \mathrm{NADPH_{nm}}$
135 $\nu\mathrm{GDH}^{\mathrm{r}}(n)$	$\mathrm{AKG_{nm}} + \mathrm{NADPH_{nm}} \rightarrow \mathrm{GLU_{nm}} + \mathrm{NADP_{nm}}$
136 $\nu\mathrm{GDH}^{\mathrm{f}}(a)$	$\mathrm{GLU_{am}} + \mathrm{NADP_{am}} \rightarrow \mathrm{AKG_{am}} + \mathrm{NADPH_{am}}$
137 $\nu\mathrm{GDH}^{\mathrm{r}}(a)$	$\mathrm{AKG_{am}} + \mathrm{NADPH_{am}} \rightarrow \mathrm{GLU_{am}} + \mathrm{NADP_{am}}$

Housekeeping

138 $\nu\mathrm{ATP}(n)$	$\mathrm{ATP_{n}} \rightarrow \mathrm{ADP_{n}}$
139 $\nu\mathrm{ATP}(a)$	$\mathrm{ATP_{a}} \rightarrow \mathrm{ADP_{a}}$

Table 2: List of metabolites.

No.	*Abbr.*	*Name*	*No.*	*Abbr.*	*Name*
		**Neuron**			
1	$ACoA_n$	Acetyl-CoA	26	K_n	Potassium
2	ADP_n	Adenosine diphosphate	27	LAC_n	Lactic acid
3	AKG_{nc}	α-Ketoglutaric acid	28	MAL_{nc}	Malic acid
4	AKG_{nm}	α-Ketoglutaric acid (m)	29	MAL_{nm}	Malic acid (m)
5	AMP_n	Adenosine monophosphate	30	$NADH_{nc}$	N. adenine dinucleotide
6	ASC_n	Ascorbic acid	31	$NADH_{nm}$	N. adenine dinucleotide (m)
7	ASP_{nc}	Aspartic acid	32	NAD_{nc}	N. adenine dinucleotide
8	ASP_{nm}	Aspartic acid (m)	33	NAD_{nm}	N. adenine dinucleotide (m)
9	ATP_n	Adenosine triphosphate	34	$NADPH_n$	N. adenine dinucleotideph.
10	BPG_n	1,3-Bisphosphoglyceric acid	35	$NADPH_{nc}$	N. adenine dinucleotideph.
11	CIT_n	Citrate	36	$NADPH_{nm}$	N. adenine dinucleotideph.(m)
12	CoA_n	Coenzyme A	37	$NADP_n$	N. adenine dinucleotideph.
13	Cr_n	Creatine	38	$NADP_{nm}$	N. adenine dinucleotideph.(m)
14	DHA_n	Dehydroascorbic acid	39	Na_n	Sodium
15	$F6P_n$	Fructose 6-phosphate	40	$O2_n$	Oxygen
16	FUM_n	Fumaric acid	41	OAA_{nc}	Oxaloacetic acid
17	$G6P_n$	Glucose 6-phosphate	42	OAA_{nm}	Oxaloacetic acid
18	GAP_n	Glyceraldehyde 3-phosphate	43	PCr_n	Phosphocreatine
19	GLC_n	Glucose	44	PEP_n	Phosphoenolpyruvic acid
20	GLN_n	Glutamine	45	PYR_{nc}	Pyruvic acid
21	GLU_{nc}	Glutamate	46	PYR_{nm}	Pyruvic acid (m)
22	GLU_{nm}	Glutamate (m)	47	ROS_n	Reactive oxygen species
23	GLU_{nv}	Glutamate (vescicle)	48	$SCoA_n$	Succinyl-CoA
24	GSH_n	Glutathione	49	SUC_n	Succinic acid
25	$GSSG_n$	Glutathione disulfide			

Table 2: List of metabolites.

No.	*Abbr.*	*Name*	*No.*	*Abbr.*	*Name*
		**Astrocyte**			
50	$ACoA_a$	Acetyl-CoA	74	K_a	Potassium
51	ADP_a	denosine diphosphate	75	LAC_a	Lactic acid
52	AKG_{ac}	α-Ketoglutaric acid	76	MAL_{ac}	Malic acid
53	AKG_{am}	α-Ketoglutaric acid (m)	77	MAL_{am}	Malic acid (m)
54	AMP_a	Adenosine monophosphate	78	Na_a	Sodium
55	ASC_a	Ascorbic acid	79	NAD_{ac}	N. adenine dinucleotide
56	ASP_{ac}	Aspartic acid	80	NAD_{am}	N. adenine dinucleotide (m)
57	ASP_{am}	Aspartic acid (m)	81	$NADH_{ac}$	N. adenine dinucleotide
58	ATP_a	Adenosine triphosphate	82	$NADH_{am}$	N. adenine dinucleotide (m)
59	BPG_a	1,3-Bisphosphoglyceric acid	83	$NADP_a$	N. adenine dinucleotideph.
60	CIT_a	Citrate	84	$NADP_{am}$	N. adenine dinucleotideph. (m)
61	CoA_a	Coenzyme A	85	$NADPH_a$	N. adenine dinucleotideph.
62	Cr_a	Creatine	86	$NADPH_{ac}$	N. adenine dinucleotideph.
63	DHA_a	Dehydroascorbic acid	87	$NADPH_{am}$	N. adenine dinucleotideph. (m)
64	$F6P_a$	Fructose 6-phosphate	88	$O2_a$	Oxygen
65	FUM_a	Fumaric acid	89	OAA_{ac}	Oxaloacetic acid
66	$G6P_a$	Glucose 6-phosphate	90	OAA_{am}	Oxaloacetic acid (m)
67	GAP_a	Glyceraldehyde 3-phosphate	91	PCr_a	Phosphocreatine
68	GLC_a	Glucose	92	PEP_a	Phosphoenolpyruvic acid
69	GLN_a	Glutamine	93	PYR_{ac}	Pyruvic acid
70	GLU_{ac}	Glutamate	94	PYR_{am}	Pyruvic acid (m)
71	GLU_{am}	Glutamate (m)	95	ROS_a	Reactive oxygen species
72	GSH_a	Glutathione	96	$SCoA_a$	Succinyl-CoA
73	$GSSG_a$	Glutathione disulfide	97	SUC_a	Succinic acid
		**Extracellular space and blood capillary**			
98	ASC_e	Ascorbic acid	104	LAC_e	Lactic acid
99	DHA_e	Dehydroascorbic acid	105	Na_e	Sodium
100	GLC_e	Glucose	106	GLC_c	Glucose
101	GLU_e	Glutamate	107	LAC_c	Lactic acid
102	GSH_e	Glutathione	108	$O2_c$	Oxygen
103	K_e	Potassium			

References

1. Jamshidi, Edwards J, Fahland T, Church B, Palsson B (2001) Dynamic simulation of the human red blood cell metabolic network. Bioinformatics 17: 286–287

2. Varma A, PalssonB (1994) Metabolic Flux Balancing: Basic Concepts, Scientific and Practical Use. Nature Biotech 12: 994–998

3. Kauffman K, Prakash P, Edwards J (2003) Advances in flux balance analysis. Curr Opin Biotech 14: 491–496

4. Lee JM, Gianchandani E, Papin J (2006) Flux balance analysis in the era of metabolomics. Brief Bioinf 7: 140–150

5. Palsson B (2006) *Systems biology: Properties of reconstructed networks* (Cambridge University Press)

6. Beard DA, Qian H (2008) *Chemical Biophysics: Quantitative Analysis of Cellular Systems* (Cambridge Univesity Press)

7. Oberhardt M, Chavali A, Papin J (2009) Flux balance analysis: interrogating genome-scale metabolic networks. Meth Mol Biol 500: 61–80

8. Orth J, Thiele I, Palsson B (2010) What is flux balance analysis? Nature Biotech 28: 245–248

9. Price N, Schellenberger J and Palsson B (2004) Uniform Sampling of Steady-State Flux Spaces: Means to Design Experiments and to Interpret Enzymopathies. Biophys J 87: 2172–2186

10. Braunstein A, Mulet R, Pagnani A (2008) Estimating the size of the solution space of metabolic networks. BMC Bioinformatics 9: 240

11. Varma A, Palsson B (1994) Stoichiometric flux balance models quantitatively predict growth and metabolic by-product secretion in wild-type Escherichia coli W3110. Appl Environ Microbiol 60: 3724–3731

12. Edwards J, Covert M, Palsson B (2002) Metabolic modelling of microbes: the flux-balance approach. Environ Microbiol 4: 133–140

13. Feist A, Palsson B (2008) The growing scope of applications of genome-scale metabolic reconstructions using *Escherichia coli*. Nature Biotech 26: 659–667

14. Lewis NE, Hixson KK, Conrad TM, Lerman JA, Charusanti P, Polpitiya AD, Adkins JN, Schramm G, Purvine SO, Lopez-Ferrer D, Weitz KK, Eils R, König R, Smith RD, Palsson B (2010) Omic data from evolved E. coli are consistent with computed optimal growth from genome-scale models. Mol Sys Biol 6: 390

15. Gale D (1960) *The theory of linear economic models* (The University of Chicago Press)

16. Imielinski M, Belta C, Halasz A, Rubin H (2005) Investigating metabolite essentiality through genome-scale analysis of Escherichia coli production capabilities. Bioinformatics 21: 2008–2016

17. Imielinski M, Belta C, Rubin H, Halasz A (2006) Systematic Analysis of Conservation Relations in Escherichia coli Genome-Scale Metabolic Network Reveals Novel Growth Media. Biophys J 90: 2659–2672

18. Warren PB, Jones JL (2007) Duality, thermodynamics, and the linear programming problem in constraint-based models of metabolism. Phys Rev Lett 99: 108101

19. De Martino D, Figliuzzi M, De Martino A, Marinari E (2012) A Scalable Algorithm to Explore the Gibbs Energy Landscape of Genome-Scale Metabolic Networks. PLoS Comput Biol 8(6): e1002562.

20. De Martino A, Marsili M (2005) Typical properties of optimal growth in the Von Neumann expanding model for large random economies. J Stat Mech L09003

21. De Martino A, Martelli C, Monasson R, Perez Castillo I (2007) Von Neumann's expanding model on random graphs. J Stat Mech P05012

22. De Martino A, Martelli C, Massucci FA (2009) On the role of conserved moieties in shaping the robustness and production capabilities of reaction networks. Europhys Lett 85: 38007

23. De Martino A, Figliuzzi M and Marsili M (2010) One way to grow, many ways to shrink: the reversible Von Neumann expanding model. J Stat Mech P07032

24. Schrijver A (1986) *Theory of linear and integer programming.* (John Wiley & Sons Ltd)

25. Krauth W, Mezard M (1987) Learning algorithms with optimal stability in neural networks. J Phys A 20: L745–L752

26. Martelli C, De Martino A, Marinari E, Marsili M, Perez Castillo I (2009) Identifying essential genes in Escherichia coli from a metabolic optimization principle. Proc Nat Acad Sci USA 106: 2607–2611

27. De Martino A, Granata D, Marinari E, Martelli C, Van Kerrebroeck V (2010) Optimal Fluxes, Reaction Replaceability, and Response to Enzymopathies in the Human Red Blood Cell. J Biomed Biotech 2010: 415148

28. De Martino A, Marinari E (2010) The solution space of metabolic networks: producibility, robustness and fluctuations. J Phys (Conf Ser) 233: 012019
